# Mechanistic insight of curcumin: a potential pharmacological candidate for epilepsy

**DOI:** 10.3389/fphar.2024.1531288

**Published:** 2025-01-07

**Authors:** Saima Khatoon, Nida Kalam

**Affiliations:** ^1^ Department of Obstetrics, Gynecology and Reproductive Sciences, School of Medicine, University of Maryland, Baltimore, MD, United States; ^2^ Infection and Immunity Research Strength, Jeffrey Cheah School of Medicine and Health Sciences, Monash University, Bandar Sunway, Malaysia

**Keywords:** curcumin, epilepsy, inflammation, apoptosis, cognition, oxidative stress, epigenetics, nanocarrier systems

## Abstract

Recurrent spontaneous seizures with an extended epileptic discharge are the hallmarks of epilepsy. At present, there are several available anti-epileptic drugs (AEDs) in the market. Still no adequate treatment for epilepsy treatment is available. The main disadvantages of AEDs are their associated adverse effects. It is a challenge to develop new therapies that can reduce seizures by modulating the underlying mechanisms with no adverse effects. In the last decade, the neuromodulatory potential of phytoconstituents has sparked their usage in the treatment of central nervous system disorders. Curcumin is an active polyphenolic component that interacts at cellular and molecular levels. Curcumin’s neuroprotective properties have been discovered in recent preclinical and clinical studies due to its immunomodulatory effects. Curcumin has the propensity to modulate signaling pathways involved in cell survival and manage oxidative stress, apoptosis, and inflammatory mechanisms. Further, curcumin can persuade epigenetic alterations, including histone modifications (acetylation/deacetylation), which are the changes responsible for the altered expression of genes facilitating the process of epileptogenesis. The bioavailability of curcumin in the brain is a concern that needs to be tackled. Therefore, nanonization has emerged as a novel drug delivery system to enhance the pharmacokinetics of curcumin. In the present review, we reviewed curcumin’s modulatory effects on potential biomarkers involved in epileptogenesis including dendritic cells, T cell subsets, cytokines, chemokines, apoptosis mediators, antioxidant mechanisms, and cognition impairment. Also, we have discussed the nanocarrier systems for encapsulating curcumin, offering a promising approach to enhance bioavailability of curcumin.

## 1 Introduction

About 70 million people worldwide suffer from epilepsy, a chronic brain disease that is considered to be one of the most common neurological disorders; 80% of those affected live in developing nations (Khatoon et al., 2021). Epilepsy is a disorder of the brain characterized by repeated electrical activity caused by dysregulation of the brain’s excitatory and inhibitory mechanisms (Dua et al., 2006). Seizures typically start in confined areas of the brain and can either stay localized known as “focal,” or spread to other brain parts, known as “generalized” seizures. For years, this disease was thought to be communicable and associated with societal stigmas, earning the label “spiritual illness.” Even though the blinds were eventually removed, the sickness remains a mystery. It is one of the most prevalent severe neurological illnesses and afflicts nearly 1% of the world’s population, necessitating never-ending research to unravel underlying pathomechanisms (Gangar and Bhatt, 2020). According to the studies, epilepsy poses a major burden in the form of seizure-associated disability, fatalities, comorbidities, stigmatization, and major financial costs. Significant advances have been made in the previous decade or two, paving the way for an elaborative understanding of the pathomechanisms that form the basis of disease and contribute to its prognosis (Moshé et al., 2015). Epilepsy still has no definitive cure, making treatment challenging, and almost one-third of persons with epilepsy are resistant to present clinically approved medications. Currently, medications in clinical use focus on symptom alleviation rather than addressing the cause at the cellular and molecular levels. Furthermore, various anti-epileptic drugs (AEDs) developed in the recent 2 decades, with a mixed effect on the percentage of patients who obtained total seizure freedom. AEDs such as phenobarbital, phenytoin, levetiracetam, and lamotrigine have been proven to be P-gp substrates in previous investigations (Luna-Tortós et al., 2008). Furthermore, the upregulation of P-gp has been linked to drug-resistant epilepsy in both epileptic individuals and epileptic animals (Chen et al., 2017). In recent years, explorative studies focused on the molecular signaling pathways, but a shift in the focus from combating pharmacoresistance is required from the exploration for medications that decrease the symptoms (seizures) in favour of a focus on new treatments that target the primary disease.

The linked side effects often cause AEDs treatment failures. They not only cause patients to discontinue therapy early, but they also prevent patients from receiving therapeutic doses and have a detrimental impact on the adherence of patients (Toledano and Gil-Nagel, 2008). Furthermore, anti-epileptic drug side effects may cause associated disabilities, comorbidities, and death (Gilliam et al., 2004). Older medications like primidone, phenobarbital, and benzodiazepines have sedative effects ranging from insignificant drowsiness or exhaustion to profound lethargy. Drowsiness, jerking movements, vertigo, ataxia, gait impairments, vision changes, nystagmus, and tremors are the symptoms of poor coordination. All first-generation AEDs, including phenytoin, carbamazepine, primidone, and benzodiazepines, have a high risk of causing coordination problems. Second-generation anti-epileptic medicines, on the other hand, can cause these side effects (Walia et al., 2004). About 15–20 percent of epilepsy patients who take anti-epileptic medicines experience negative psychological consequences (Chen et al., 2017; Perucca and Gilliam, 2012). These side effects include irritation, aggressiveness, agitation, and violent behavior, as well as depression and psychosis (Perucca and Gilliam, 2012).

Idiosyncratic reactions are one of the concerns because they can cause life-threatening side effects and in severe cases of reaction it necessitates therapy termination. Cellular damage caused by the chemical agent or its by-product, drug, or pharmaceutically active ingredient association with unusual interaction with targets present in the host is among the processes that underpin idiosyncratic reactions associated with AEDs therapy (Zaccara et al., 2007). Furthermore, despite the availability of numerous AEDs, individuals with mesial temporal lobe epilepsy do not attain total seizure independence, and therapy with third-generation AEDs does not obviate the need for surgery (Pohlen et al., 2017). Some biologics, such as monoclonal antibodies, have been used to treat epilepsy; however, the mechanisms underlying human epileptogenesis are still unknown, and clinically meaningful targets for therapy development are still hypothetical (Zhao et al., 2017). Pharmacoresistant epilepsy is the most severe issue that requires immediate attention. The term “resistant epilepsy” is described as the “failure of adequate trials of two tolerated and correctly planned AED regimens (whether as single drug therapies or in combination) to attain seizure independence,” according to the International League against Epilepsy (Weaver and Pohlmann-Eden, 2013). It is a condition in which almost 30% of epilepsy patients develop frequent seizures the despite treatment and this condition is labelled by three interchangeable phrases, known as “pharmacoresistant epilepsy,” medically intractable epilepsy, or refractory epilepsy (Stafstrom and Carmant, 2015). This form of epilepsy is characterized as chronic, irreversible disorder that is also linked to increasing psychosocial and physical morbidity, as well as a high mortality rate (Englot et al., 2017). Though surgery remains a promising therapy option for pharmacoresistant epilepsy, the risk of losing brain functions renders it unsuitable for most patients. Non-pharmacological therapies such as targeted cooling, vagus nerve stimulation, and gamma knife therapy are also becoming more popular (Rocha, 2013). More than half a million patients with neurological problems have had radiosurgery with Gamma Knife, which offers a reliable, noninvasive treatment for intracranial pathology. Ionising radiation, or radiation that may remove electrons from atoms or molecules and cause chemical bonds to break, new bonds to form, or free radicals to be produced, is essential to radiosurgery. Ionising radiation breaks cellular DNA in cases of radiosurgery of the brain. Substantial percentage of individuals experience seizure independence when only patients who got high-dose radiation were studied (Rolston et al., 2011).

As a result, there is a demand for alternative drugs and therapies, which primarily involves medical techniques, change in lifestyle, and the administration of synthetic and natural medicinal moieties.

The two primary and most well-known databases for biomedical literature, PubMed and Google Scholar, were used to prepare the review. Research Gate and PubMed’s electronic resources were used to access full-text documents. The most recent findings are included in the review’s content, which references recent articles. Immunopathology of epilepsy, epilepsy and oxidative stress, epilepsy and cognitive impairment, curcumin and neurological disorders, curcumin and oxidative stress, curcumin and cognitive impairment, curcumin and epigenetics, and novel drug delivery systems for curcumin were the terms and phrases that were searched for. The chosen research and review articles were picked because they were closely related to the selected topic.

## 2 Current treatment strategy for epilepsy

With the advent of several novel anti-epileptic treatments as well as better formulations of older treatments, epilepsy pharmacotherapy has made tremendous strides in the past few years. Since 1851, many first-generation AEDs have been developed for the treatment of epilepsy. Some of the examples of first-generation AEDs including, primidone, carbamazepine, sodium valproate, phenobarbitone, phenytoin, ethosuximide, and phenobarbitone. Later, second-generation AEDs have been developed, and some the examples, including, lacosamide, gabapentin, lamotrigine, oxcarbazepine, levetiracetam, topiramate, and zonisamide. In recent decades, novel AEDs with distinctive modes of action, including perampanel, retigabine, and brivaracetam, have been released on the market. The multiple processes by which these approved AEDs for the treatment of epilepsy function primarily involve the modulation of voltage-dependent ion channels, activation of GABA, and inhibition of glutamate receptors. Even in patients who react well to treatment, current anti-epileptic drugs have no impact on the inherent natural history or progression of the condition. Additionally, there are not any drugs on the market right now that can stop epilepsy from arising from following a head injury. It is projected that the rapid advancement of understanding of cellular, molecular, and genetic causes of epilepsy will result in more potent and prominent treatments, and preventative measures, and might cures various forms of epilepsy. Managing the condition through patient care and developing various therapeutic strategies is one of the key coping mechanisms. Numerous studies and research findings indicate that combining multiple medications can help control the disease. Different dosages of the same medications have been observed to benefit various individuals. Lamotrigine and sodium valproate has been shown to help treat partial-onset and generalized seizures in several animal models. Other commonly advised combinations include lamotrigine and topiramate for treating a variety of seizures and valproate with ethosuximide for managing absence seizures (Kwan et al., 2011; Brodie and Yuen, 1997).

Surgery is a treatment option for patients with DRE (drug-refractory epilepsy), particularly if they have a condition that can be treated surgically, such as unilateral hippocampal sclerosis or other treatable abnormalities. Therefore, after considering additional anti-epileptic drug trials, various surgical procedures can be carried out based on the indication to manage and treat seizures (Spencer and Huh, 2008). The ketogenic diet is employed as a treatment technique in children with DRE. They are also offered as newborn ketogenic diets based on formula (Kwan et al., 2011). Many different forms of seizures appear to be easier to manage with a ketogenic diet. A ketogenic diet is recognized for controlling gene expression via the epigenetic process. It has been discovered that dietary methyl donor consumption, such as choline, can significantly impact the DNA methylation process. Children with refractory seizures are often treated with ketogenic diets high in fat and low in carbohydrates (Boison and Rho, 2020).

Moreover, studies on micronutrient deficiencies, such as vitamin B12 deficiency, suggest that they may be involved in the pathway and may be helpful in future therapeutic approaches (Sinha et al., 2014). A device known as the vagus nerve stimulator has been authorized for use in adults and adolescents with partial-onset seizures refractory to anti-epileptic medications (Neal et al., 2008). The patient has a vagus nerve stimulator inserted in the chest area that produces a pulse and sends an electrical current to the neck’s vagus nerve (Milby et al., 2008).

Combinations of anti-inflammatory medications that target many pathways may be expected to be more successful than individual medications alone due to the complexity of the inflammatory processes that are linked to epileptogenesis. According to research in teenage rats, treatment with anakinra plus a COX-2 inhibitor during epileptogenesis reduced chronic seizures and neuronal cell death, but not treatment with either medication alone (Kwon et al., 2013). Similar to this, when given to animals for a short period of time soon after the onset of epilepsy, VX-765 and an experimental TLR4 antagonist effectively halted the disease’s progression and reduced 90% of recurrent seizures (Iori et al., 2017). During epileptogenesis, blocking only one inflammatory route imposed non-significant impact (Iori et al., 2013). Additionally, a conjunction of the ketogenic diet and MAGL inhibition in the acute stage of status epilepticus in mice produced more profound benefits than either intervention alone in stopping persistent epileptic hyperactivity (Terrone et al., 2018); additionally, in a mouse experiment, anakinra improved the ability of diazepam to shorten the period of status epilepticus (Xu et al., 2016).

### 2.1 Disease modifying therapies in epilepsy

Neuroinflammation, neuronal damage, synaptic reorganisation, and genetic abnormalities are some of the factors that frequently lead to epilepsy. In patients who are at risk (such as those who have had a stroke, infection, or traumatic brain damage), DMTs would try to prevent the start of epilepsy or alter the course of the disease to lessen the frequency and intensity of seizures (Kwon et al., 2013). DMTs may include approaches for avoiding neuronal damage or death, which are frequent causes of epilepsy. Examples include substances that target excitotoxicity, mitochondrial malfunction, or oxidative stress (Khatri and Juvekar, 2016). Epileptogenesis, or the development of epilepsy, can result from persistent inflammation in the brain. Anakinra and other cytokine inhibitors are examples of medications that target inflammatory pathways like IL-1β (Dilena et al., 2019). Epilepsy is thought to develop as a result of epigenetic modifications. For instance, inhibitors of histone deacetylase (HDAC) change the expression of genes to stop epileptogenesis (Teafatiller et al., 2022). When it comes to monogenic epilepsies, CRISPR technology or viral vectors can be used to compensate for or correct gene alterations such as SCN1A (Dravet syndrome) (Carpenter and Lignani, 2021). Recent developments in antisense oligonucleotides for hereditary epilepsies are one example (Li et al., 2021a). Some epilepsies are characterised by synaptic reorganisation, such as the emergence of mossy fibre in the hippocampus. In order to stop progression, potential treatments might interfere with maladaptive synaptic alterations (Danzer, 2017). DMTs might concentrate on stopping tumour growth in situations of epilepsy brought on by tumours (such as low-grade gliomas) (Seidel et al., 2022).

In order to target the fundamental causes of epilepsy instead of merely suppressing seizures, potential disease-modifying medicines are being actively investigated. Anti-inflammatory drugs that target neuroinflammation, including COX-2 inhibitors or IL-1 receptor antagonists (like anakinra), are being researched for their potential to prevent post-trauma epilepsy. Neurosteroids may assist stabilise neuronal excitability and provide long-term advantages in modulating the progression of disease. One such neurosteroid is ganaxolone, a synthetic analogue of allopregnanolone.

For epilepsy linked to tuberous sclerosis complex (TSC), mTOR inhibitors, such everolimus, are already authorised and have demonstrated potential in changing the progression of the condition. Levetiracetam and other preventative medications are also being researched for their ability to stop epilepsy after brain trauma. For certain genetic epilepsies, like Dravet syndrome and Angelman syndrome, new gene-targeted treatments, such as antisense oligonucleotides (ASOs), are being developed. These therapies provide a focused approach to disease modification.

### 2.2 Pharmacoresistance in epilepsy

A pharmacokinetic theory states that the overexpression of drug efflux vectors in peripheral organs results in a decrease in AED levels. This prevents medications with high enough concentrations from entering the brain and reaching the epileptic centre. This hypothesis is supported by clinical observations in which the overexpression of P-glycoprotein (Pgp), multidrug resistance protein 1, or other transporters on the blood-brain barrier and in neurons was unable to account for the decline in ASM concentration (Tang et al., 2017). The transport hypothesis states that drug efflux carrier upregulation in medication-resistant epilepsy takes place directly in the blood-brain barrier rather than in its periphery, which reduces drug absorption by the brain and, thus, causes resistance (Tang et al., 2017). One of the basic mechanisms of cognition, perception, and awareness is the alteration of the neural network. Disturbances in network activity are essential to the pathophysiology of brain disorders. Individual brain models derived from diffuse magnetic resonance imaging of fifteen individuals with drug-resistant epilepsy have been demonstrated to have prognostic power (Proix et al., 2017). Drug-resistant epilepsy is frequently linked to cortical dysplasia (Barkovich et al., 2015). According to the intrinsic severity theory, both medication resistance and the severity of epilepsy are influenced by common neurobiological processes. Hippocampal tissues taken from patients with mesial temporal lobe epilepsy, the most prevalent type of focal epilepsy that affects 40% of adults and is 30% resistant to AEDs, were subjected to a transcriptome study. Three significant gene clusters that are mostly linked to neuroinflammation and innate immunity, synaptic transmission, and neural network regulation were found to have abnormal expression in this investigation (Bazhanova et al., 2021). Different forms of epilepsy have been linked to polymorphisms in genes that encode channels, receptors, transporters, synaptic transmission, etc. Some of these polymorphisms have also been linked to refractory epilepsy (Liu et al., 2016).

### 2.3 Curcumin

Curcumin is a phenolic compound with enormous benefits and immense potential in treating various pathological conditions. Characteristically, it has a bright coloured lipophilic polyphenolic chemical obtained from the rhizome of the turmeric plant (Curcumin longa), which is a tropical Southeast Asian spice. Turmeric powder, which contains 2%–5% curcumin, has been used as an anti-inflammatory medication in Indian and Chinese traditional medicine for millennia. It coexists with its keto-enol tautomeric forms in a state of equilibrium. It is partially soluble in water and not very stable; however, it degrades more quickly in a basic medium. Curcumin poses multiple benefits, including, antioxidant, anti-cancer, anti-arthritic, anti-microbial, anti-diabetic, and anti-inflammatory activities, which can be used to treat a variety of ailments including tendinitis, liver cirrhosis, Alzheimer’s disease, epilepsy, cardiovascular disease, low blood sugar, gastrointestinal issues, worms, inflammation, cancer, epidermis, and ocular infections. Additionally, it has shown tendency to cross the blood-brain barrier and demonstrated its protective efficacy in various neurological diseases (Eghbaliferiz et al., 2020).

**FIGURE 1:**
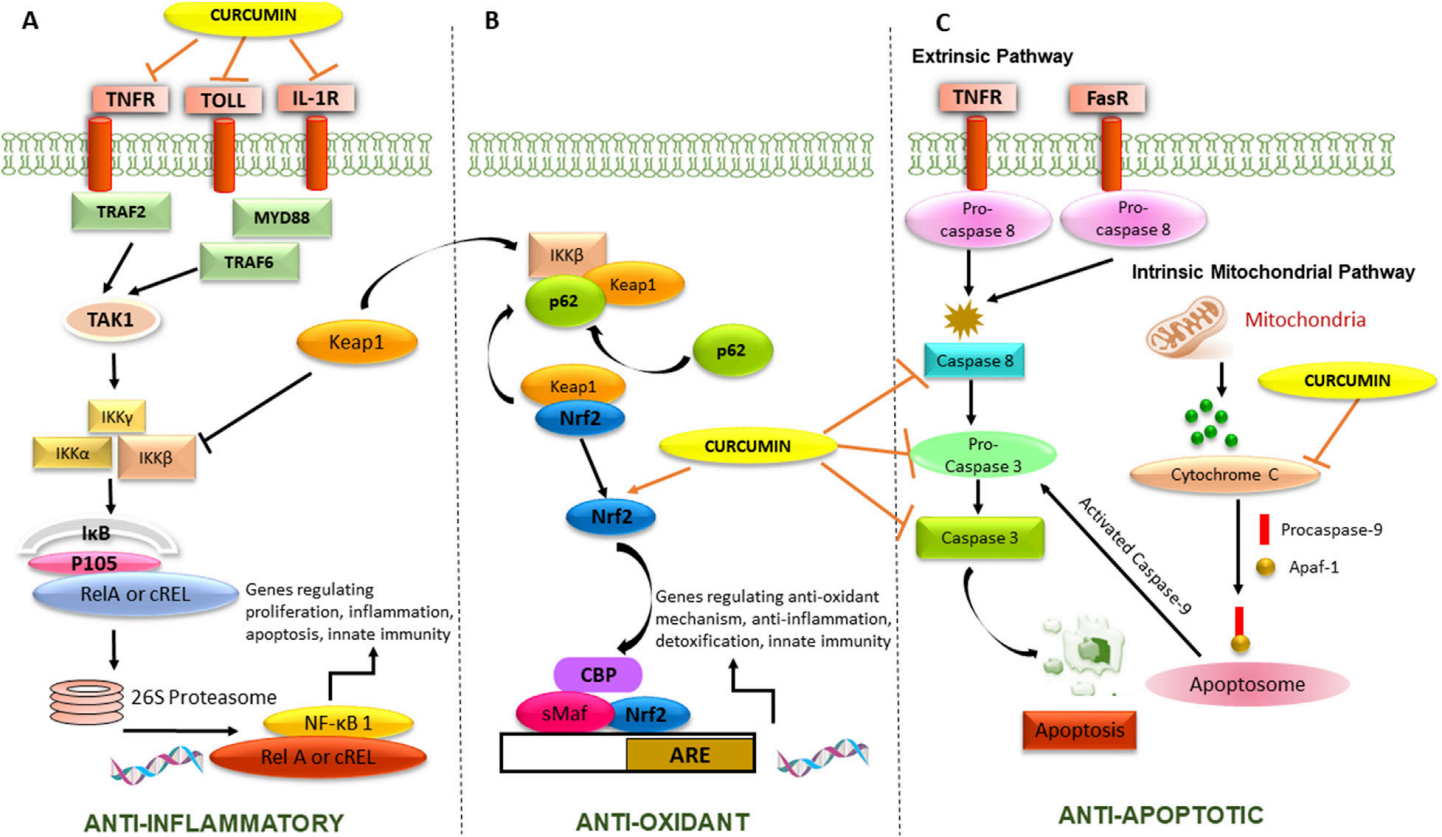
Mechanism of action of curcumin responsible for its anti-epileptic activity. The Figure illustrates **(A)** Anti-inflammatory activity by inhibiting cell surface receptors and NF-κB transcription factor, playing a pivotal role in regulating cytokines and chemokines, **(B)** Antioxidant activity by potentiating Nrf2-ARE pathway responsible for transcription of antioxidant genes, **(C)** Anti-apoptotic activity by inhibiting extrinsic and intrinsic mitochondrial pathway.

The modulatory effect of curcumin on some of the cell signaling pathways responsible for its disease-modifying properties, represents in Figure 1. Curcumin has been found to modulate Toll-like receptors (TLRs) and the nuclear factor-kappa B (NF-κB) pathway, which are both involved in the innate immune response and inflammation. Curcumin interrupts the activation of TLR signaling by downregulating the expression of TLRs and inhibiting its downstream signaling molecules, like MyD88, and IRAK. Downregulation of TLR signaling can lead to reduction of proinflammatory cytokine and chemokine release, thus reduce the inflammatory cascade release.

Curcumin can inhibit the activation of NF-κB by blocking the phosphorylation and degradation of its inhibitor, IκBα, which usually leads to the nuclear translocation of NF-κB and the activation of its target genes. By inhibiting NF-κB activation, curcumin can reduce the expression of pro-inflammatory cytokines, chemokines, and other inflammatory mediators. Curcumin has also been found to modulate the nuclear factor erythroid 2-related factor 2-antioxidant response element (Nrf2-ARE) pathway, which is involved in the regulation of cellular antioxidant and detoxification responses. Curcumin has been found to have complex effects on the apoptosis signaling pathway. In terms of the extrinsic and intrinsic pathways, curcumin has been reported to modulate both pathways.

According to Nutraceutical Bioavailability Classification Scheme curcumin exhibits poor bioaccessibility, but it is fairly soluble in gastrointestinal fluids and unstable to alkaline pH. Due to its diketone moiety, curcumin often occurs in the solid phase and the keto form in acidic and neutral environments. Curcumin has limited stability at pH = 7 and weak solubility in water. Furthermore, curcumin has a low bioavailability. In fact, it is quickly metabolized in the gut, where it undergoes substantial biotransformation before being quickly eliminated (Xie et al., 2011; Nabavi et al., 2018). These variables collectively cause the concentration of this molecule in human plasma to be at nanomolar levels, which restricts its biological functions. The detailed mechanism of action of curcumin in inhibiting the process of epileptogenesis will be discussed in the following sections.

### 2.4 Effect of curcumin on neurological disorders

Curcumin extensively studied for its potential neuroprotective effects and potential as a therapeutic agent in treating neurological diseases. One of the most studied neurological diseases with curcumin is Alzheimer’s disease (AD). Curcumin has been shown to have various potential neuroprotective effects in AD, including reducing the accumulation of β-amyloid (βA) plaques, decreasing neuroinflammation, and reducing oxidative stress. Studies in animal models of AD have shown that curcumin can improve cognitive function and memory, reducing the disease’s pathological features (Lin et al., 2022). Curcumin also aids in preserving mitochondria from the damaging effects of AD; for example, it lessens the oxidative stress brought on by βA in PC12 and the levels of IL-1β and oxidized proteins in the brain of AD mice (Kim et al., 2001; Lim et al., 2001).

Curcumin extensively studied for its potential therapeutic effects in other neurological diseases, including Parkinson’s disease (PD), multiple sclerosis (MS), Huntington’s disease, and depression.

Treatment with 1-methyl-4-phenyl-1,2,3,6-tetrahydropyridine (MPTP) increased the heat shock protein 90 (HSP90) level in SH-SY5Y dopaminergic cells, but curcumin restored this effect. HSP90’s overexpression boosted the effects of curcumin on PD, most likely through the elevation of HSP90. HSP90’s silencing considerably reduced the effect caused by curcumin; on the other hand, it enhanced its benefit (Sang et al., 2018). Curcumin reversed motor impairments in the rotenone-PD rat model and boosted the activity of antioxidant enzymes that underpin it is *in-vivo* antioxidant capacity, possibly acting in a neuroprotective way (Khatri and Juvekar, 2016). Moreover, it reversed hippocampal synaptic changes (Darbinyan et al., 2017). Lewis bodies’ primary constituent, α-Syn, can bind to curcumin and prevent it from accumulating in neuronal cells (Ahmad and Lapidus, 2012).

The autoimmune encephalomyelitis model for MS (multiple sclerosis) was used as animal models for investigation of effect of curcumin in MS. Reportedly, curcumin inhibited cytokines, IFNγ, IL-17, and IL-12 family members (Kanakasabai et al., 2012). These findings are consistent with those from a related rat model in which curcumin treatment enhanced the degree of myelination, most likely via restoring iNOS mRNA expression and amplifying the Nrf2 cellular defense system against oxidative damage (Mohajeri et al., 2015). Additionally, curcumin increased all the markers for oligodendrocyte progenitors and neural stem cells, including nestin (a marker for NSCs), Olig2, platelet-derived growth factor receptor (PDGFR), and brain-derived neurotrophic factor (BDNF) and nerve growth factor (NGF) (Mohajeri et al., 2015).

Micelle nanoformulation of curcumin with dose of 80 mg/day was investigated in a human clinical studies and demonstrated to decrease Th17 cells population in the peripheral circulation post 6 m months after 6 months, 80 mg/day (Dolati et al., 2018). The study also examined the effect of curcumin Th17 cells because its association with elevated levels of IL-23 and IL-17A as well as increased expression of retinoic acid-related orphan receptor γ (RORγ) in MS patients. This study also reported that treatment with micelle formulation of curcumin treatments significantly decreased RORγ mRNA levels and decreased IL-17 secretion but did not affect the expression profiles and concentration of IL-23 mRNA (Dolati et al., 2018).

NSC-34 cells infected with TDP-43 were used as Amyotrophic lateral sclerosis (ALS) cellular model to evaluate the effect of curcumin treatment in ALS in reversing the impairment caused by the overexpression of TDP-43. It was observed that uncoupling protein-2 levels were significantly reduced due to curcumin produced a beneficial effect on mitochondrial membrane potential (Lu et al., 2012). The initiation and spread of action potentials (APs), facilitated by the overexpression of TDP-43, are also a part of curcumin’s protective actions (Dong et al., 2014). Post 1 year of curcumin treatment, an ALS clinical trial utilizing nanocurcumin as an adjunctive therapy to riluzole demonstrated discovered a noticeably higher survival rate (Ahmadi et al., 2018).

The progression of post-ischemic neurodegeneration was seen to be prevented by curcumin treatment utilizing a middle cerebral artery occlusion (MCAO) in a rodent model (Pluta et al., 2015), decreased infarct volume (Zhao et al., 2010; Funk et al., 2013) and oedema of the brain at various times intervals and can achieve improved neurological scores (Zhao et al., 2010; Funk et al., 2013). The reduction of oxidative stress probably brings about these results. Moreover, curcumin decreased neuronal apoptosis by increasing the anti-apoptotic Bcl2 protein and decreasing the translocation of cytochrome-c into the cytoplasm (Zhao et al., 2010; Xia et al., 2017), and reduction of mitochondrial membrane potential (Zhang et al., 2017). Also, it has been demonstrated that curcumin has a protective effect in an intrinsically hypertensive rat model and is stroke-prone, preventing the development of stroke and improving survival rates. The presence of more mitochondrial anion carrier family proteins and the physiological regulation of mitochondrial ROS production caused by curcumin is the most plausible causes of these effects (Lan et al., 2018). These findings were further supported by an *in vitro* cellular model utilizing H_2_O_2_ to mimic oxidative stress reduced by curcumin therapy (Lan et al., 2018). Therefore, this review will mainly dissect the impact of curcumin on epileptogenic processes.

### 2.5 Curcumin and its immunomodulatory effect

According to human clinical studies, curcumin is considered to be risk-free if consumed at a se of 10 g/day, and no toxicity is observed in the individuals (Aggarwal et al., 2003; Jurenka, 2009). Curcumin consist of profound activities including, antioxidant, anti-inflammatory, antibacterial, hepatoprotective, neurogenesis-inducing, chemopreventive, and chemotherapeutic action (Aggarwal et al., 2003; Roberts et al., 2017; Asteriou et al., 2018; Bianchi et al., 2018; Kadam et al., 2018; Platania et al., 2018; Sharma et al., 2018; Vucic et al., 2018). Newer research has also presented an intriguing view of curcumin’s immunomodulatory potential (Jagetia and Aggarwal, 2007; Momtazi et al., 2016). Curcumin’s immune-modulating activities are attributable to its interactions with cells that mediate immunologic reactions, such as B and T-lymphocytes, macrophages, dendritic cells, cytokines, and the transcription factors involved in cell signaling pathways. Curcumin is reported to act by regulating the pleiotropic regulator of inflammation, NF-κB, PPARγ, signal transducer and activator of transcription (STAT), activator protein-1 (AP-1), Nrf2, beta catenin, and their downstream targets in signaling pathways (Han et al., 2002; Shishodia et al., 2007; Gonzales and Orlando, 2008; Soetikno et al., 2011; Ganjali et al., 2014).

The combination of p50/NF-κB1, p52/NF-κB2, p65/RelA, c-Rel, and RelB forms the NF-κB transcription factor dimers family. These transcription factors play a key role in chronic inflammation and are implicated in development, inflammation, and immunological response (Hayden and Ghosh, 2008; Hoesel and Schmid, 2013; Ledoux and Perkins, 2014). In T-lymphocytes, NF-κB plays a vital role in the synthesis of pro-inflammatory molecules such as interleukin (IL)-16, IL-4, IL-2, interferon γ (IFN-γ) and TNF-α (Infantino et al., 2014; Valim et al., 2015; Zhang et al., 2016; Templin et al., 2017). Curcumin exerts pleiotropic effects by impeding NF-κB transcription by inhibiting I kappa B kinase-a (IKK-a) phosphorylation, degrading I kappa B alpha degradation, inhibiting phosphorylation of I kappa B serine 32, and blocking RelA nuclear translocation (Jobin et al., 1999; Grandjean-Laquerriere et al., 2002; Banik et al., 2017). B-lymphocyte stimulator (BLYS) is a cytokine produced mainly by monocytes, dendritic cells, and macrophages in the innate immune system (Nardelli et al., 2001; Bossen and Schneider, 2006). B-cell activating factor (BAFF) plays an immunological role in the immune system’s B cell arm and immunoglobulin synthesis that could influence the functioning of B cells eventually (Ng et al., 2004). BLYS and BLYSR protein expression levels are much higher in TLE patients and in tissues of rodent epilepsy models, suggesting that BAFF and BAFFR may play critical roles in regulating immunological and inflammatory responses involved with disease development (Huang et al., 2011; Ma et al., 2017). Curcumin has been suggested as a potential new therapeutic drug for autoimmune illnesses by targeting BLYS. Curcumin’s inhibitory action on the expression of BLYS is attributed to its disruption of NF-κB signaling, which decreases p65 nuclear translocation (Huang et al., 2011). Curcumin also suppresses immune function by inhibiting mammalian target of rapamycin (mTOR) pathway and subsequently downregulating cytokine production, such as IL-6 and COX-2. It has a solid ability to block the PI3K/Akt/mTOR pathway in the SH-SY5Y cell line and in the rat model of TLE (Drion et al., 2018; Zhu and Zhu, 2018).

## 3 Pathogenesis of epilepsy

### 3.1 Immunopathology of epilepsy

The adaptive immune system after pilocarpine-induced status epilepticus is reflected by a biphasic rise of CD45^+^ immune cells in the hippocampus brain parenchyma, including innate macrophages and CD3^+^ T-lymphocytes. The hippocampus macrophages have higher granularity, which indicates that they are activated (Neumann et al., 2017). Microglial cell activation has been reported to increase the expression of IL-6, IL-1β, and TNF-α (Aronica and Gorter, 2007; Aronica and Crino, 2011) and is allied with compromised blood-brain barrier integrity (Lucas et al., 2006).

Elevated CD8-positive T lymphocytes are a hallmark of several CNS disorders involving inflammation (Bernal et al., 2002; Petito et al., 2006) which can eventually cause the precipitation of epileptic seizures. This is supported by research examining the immunological profile of patients with Rasmussen’s encephalitis. Rasmussen’s encephalitis is a progressive epileptic disease that causes the affected hemisphere to be destroyed due to unihemispheric lymphocytic infiltrates, microglial nodules, and neuronal death. T-lymphocyte fraction of brain parenchyma was primarily composed of CD8^+^ cells. CD4^+^ cells is reported to concentrate in the perivascular region of blood vessels rather than moving into the brain parenchyma. In addition, a cytotoxic T-cell mechanism has been postulated to play a role in neuronal death in human brain disease (Bien et al., 2002a). Assuming infiltrating T lymphocyte density is a criterion for inflammatory processes, disease duration, and neuronal cell death are inversely associated with this attribute. T lymphocyte levels fall as the disease progresses, but neuronal death increases. However, it should be emphasized that the number of T lymphocytes in the blood is still higher than in healthy people (Bien et al., 2002b). These observations corroborate the theory that cytotoxic T-cell reaction against neuronal cells leads to its loss. According to certain studies, these T cells encompass cytotoxic granules and are located near neurons (Bauer and Bien, 2009). This research could indicate that these cells have a role in neuronal cell death. In addition to Th1 and Th2 T cells, anti-inflammatory CD4^+^CD25+Foxp3+ regulatory T cells (Tregs) and pro-inflammatory T helper 17 (Th17) cells have been discovered. Th17 cells with retinoic acid-related orphan receptor gt (RORgt) play vital roles in generating IL-17, whereas Treg cells expressing the transcription factor, Foxp3, possess anti-inflammatory activity and preserve self-component tolerance (Oukka, 2007; Krasovsky et al., 2009). Th17/Treg balance is crucial for disease progression, and is evident in various studies of different animal models and human autoimmune and inflammatory diseases (Noack and Miossec, 2014). Patients with epilepsy have reduced circulating numbers of total lymphocytes, CD4^+^ T cells, and natural killer cells, according to studies (Bauer et al., 2008). Th17/Treg imbalance has been identified as a defining hallmark of intractable epilepsy (Ni et al., 2016). Recurrent epileptic activity in the brain can be caused by various inflammatory conditions, such as viral or autoimmune disease, highlighting the importance of inflammation in epilepsy pathogenesis (Vezzani, 2014). Furthermore, prolonged seizure activity stimulates glia and causes endothelial cells to upregulate adhesion molecules, making it easier for leukocytes to extravasate (Librizzi et al., 2007).

### 3.2 Dendritic cells in epilepsy

Dendritic cells (DCs) are the antigen-presenting cells (APCs) and are the sentinels of the immune system to capture antigens or access self-proteins unusually present in the environment and present them to the surface of T cells to assemble an antigen specific immune response or elicit tolerance, acting as bridge between both the innate and adaptive immune responses. These cells have been linked to a variety of brain disorders, although their exact function is uncertain. It is intended to consider relevant material from the disciplines of immunology and neurology. T lymphocytes’ adaptive immune response is dependent on the detection of antigens presented by APCs. Small peptides in extended conformation are seen in these cells, which are produced from antigenic proteins linked to major histocompatibility complex MHC-I and MHC-II molecules. These two are presented differently because they are degraded at two different places inside the APC (Villadangos, 2001). Antigens obtained from external proteins are managed by the endocytic route via lysosomal enzymes and expressed by MHC-II. Presentation via MHC-I molecules, on the other hand, is based on cytosolic antigen recognition in the endoplasmic reticulum, which usually involves endogenous molecules, excluding a specialized condition known as “cross-presentation,” which requires the diffusion of exogenous proteins from lysosomal compartment to cytoplasm (Guermonprez et al., 2002).

Effector T-cells can develop from naive T cells that detect peptides linked to MHC molecules. Central and peripheral tolerance mechanisms ensure the eradication of anti-self-reactive T-cells. T-cell anergy, apoptosis, or Tregs ensure tolerance to self-antigen when immature APCs present self-antigen to T cells in the lymph nodes (Starr et al., 2003; Torres-Aguilar et al., 2010). Non-lymphoid peripheral organs may have more peptides released by MHC molecules than lymphoid organs (Clement and Santambrogio, 2013). They might play a role in the onset and development of autoimmune diseases (Collado et al., 2013). The situation of the interactions between T cells and APCs dictates whether naive T cells are primed or tolerated (Steinman, 2003). As a result, APCs play an essential role in tolerance pathomechanisms, and the characteristics of these cells and their surrounding environment influence whether tolerance or immunity is generated. DCs and T-cells interact via CD40:CD40L, resulting in the production of various cytokines and chemokines, including TNF-α, IL-1β, IL-12, type I interferon, and macrophage inflammatory protein-1 (Guermonprez et al., 2002; Banchereau and Steinman, 1998). Furthermore, the transition from immature DCs (iDCs) to mature DCs (mDCs) results in the expression of C-C chemokine receptor type 7 (CCR7) and loss of adhesion to epithelial cells, resulting in relocation towards lymph node T-cell-rich regions (Guermonprez et al., 2002; Winzler et al., 1997).

Recurrent seizures can be caused by several inflammatory conditions, such as viral or autoimmune disease, highlighting the importance of inflammation in epilepsy pathogenesis (Vezzani, 2014). Prolonged electrical activity during seizures stimulates glial cells and causes endothelial cells to upregulate adhesion molecules, allowing leukocytes to extravasate more easily (Librizzi et al., 2007). This reaction is not an associated epiphenomenon of the involved tissue as inhibition of cell infiltration can avert the process of ictogenesis (Fabene et al., 2008). Interestingly, efforts have been made to administer immunomodulatory agents to treat epilepsy (Vezzani, 2015). By secreting cytokines, altering neurotransmitter release or uptake, elevating BBB permeability, and injuring neuronal cells, inflammatory cells increase neuronal excitation and reduce seizure thresholds (Vezzani, 2014). Li et al. (2013) recently demonstrated that DCs might be detected 24 h after the production of seizures in adult rats using Li-pilocarpine-induced status epilepticus model. CD11^+^ cells were transferred from the periphery and were not derived from microglia, according to negative Iba-1 labelling and radiation experiments. In addition, after kainic acid-induced convulsions, EYFP-expressing cells were identified in the damaged hippocampus of the Cd11c/eyfp Tg animal (Bulloch et al., 2008). FCD (focal cortical dysplasia) is a disorder in which children develop chronic epilepsy due to spontaneous abnormalities in the cerebral cortex (Rhodes et al., 2007; Iyer et al., 2010).

Along with neurotransmitter imbalance, emerging data suggests that inflammatory mechanisms are involved in non-infectious epilepsy. In the tissue of patients with FCD, stimulation of microglia and macrophages has been observed (Boer et al., 2008). It is unclear if this inflammatory reaction is induced by recurrent episodes of seizures or is an inherent mechanism of FCD. DCs circulates in the blood arteries in chronic epileptic encephalopathy and FCD type II patients’ samples, together with perivascular T-lymphocytes (Rhodes et al., 2007; Iyer et al., 2010). PI3K-mTOR pathways are primarily associated with experimental epilepsy and clinical epilepsy studies (Iffland et al., 2022; Nguyen et al., 2022). In addition, through the PI3K/Akt/mTOR signaling pathway, the inflammatory response elicited by IL-1β increases seizure activity and plays a significant role in the pathophysiology of MTLE (Xiao et al., 2015). Also, as evident, the mTOR pathway controls DC’s function and maturation (Sathaliyawala et al., 2010). Since autoantibodies have been identified in several kinds of epilepsy, such as Rasmussen encephalitis, this suggests that DCs may be involved in the pathophysiology of epilepsy by preserving a persistent state of inflammation, potentially by triggering autoimmune processes (McNamara et al., 1999). Activation of the mTOR pathway in the CNS can affect neuronal signaling and excitation, axonal and dendritic morphology, the release of neurotransmitters, synaptic plasticity, cognition, and behavior (Bekinschtein et al., 2007; Jaworski et al., 2005; Tang et al., 2002). Axonal and dendritic structure, neurotransmitter production, neuroplasticity, and cognition and behavior are all influenced by mTOR activity in the CNS (Wong, 2013). In animal models of epilepsy and human tissue samples removed from epilepsy patients showed evidence of overactive mTOR signaling pathways in both hereditary and acquired epilepsies. For example, mutations in elements of the mTOR system cause many neurodevelopmental diseases with epileptic symptoms, the most known of which are TSC1/TSC2 and phosphatase and tensin homolog (PTEN) mutations. TSC is an autosomal dominant disorder characterized by cortical abnormalities such as tubers and subependymal giant cell astrocytomas in the brain caused by a heterozygous mutation either in TSC1 or TSC2 (Jülich and Sahin, 2014). TSC1 and TSC2 proteins form a complex that inhibits mTOR activity; therefore, deletion of these proteins causes hyperactive mTOR signaling, intellectual impairment, refractory epilepsy, intellectual impairment, and an autistic-like phenotype (Chu-Shore et al., 2010; Vignoli et al., 2015). PTEN is a tumour suppressor gene that also regulates cellular proliferation and survival while inhibiting mTOR activity (Bonneau and Longy, 2000). Loss of PTEN causes hyperactive mTOR signaling, seizures, and substantial intellectual and behavioral deficits, comparable to mutations in TSC1/TSC2 (Cupolillo et al., 2016).

### 3.3 Epilepsy and oxidative stress

Calcium signaling is involved in regulating and maintaining neuronal function, encompassing the release of neurotransmitters, neuronal excitation, neurite outgrowth, neuronal plasticity, transcription, and survival of neurons. The mitochondria regulate the free intracellular Ca^2+^ present in cells through various transport mechanisms and preserve Ca^2+^ homeostasis, and work as a Ca^2+^ buffer that regulates the intracellular Ca^2+^ levels; when Ca^2+^ levels get accumulated in the mitochondria, it is released in the matrix, and this progression involves oxidative stress and reduction of adenine nucleotides (Orrenius et al., 2007). Overload of mitochondrial Ca^2+^ ions causes the MPTP opening, eventually leading to necrosis because of ATP depletion or caspase-mediated apoptosis; this explains the multifarious interdependence between Ca^2+^ influx and ROS production (Martinc et al., 2012). Ca^2+^ release in the endoplasmic reticulum and initiation of the caspase-dependent apoptosis cascade through alterations in mitochondrial membrane permeability persuade damage of cells (Harrison et al., 2005; Pinton et al., 2008). Increased activation of glutamate receptors induces oxidative stress, referred to by the excitotoxicity event, and, plays a crucial role in seizure-induced damage (Epstein et al., 1994).

Generalized epilepsy is the chronic form of epilepsy that is distinguished by repeated seizures and causes excessive levels of ROS and RNS in the brain tissue; a series of clinical and preclinical research have documented the association between epileptic seizures and ROS. It is an interesting fact that it is still unclear whether oxidative stress is a basis or result of seizures; it is extensively stated that elevated free radical production can cause persistent seizure activity, which might ultimately result in dysfunction of mitochondria in the limbic structures of the brain that causes neuronal cell damage and loss during epileptogenesis (Chen et al., 2010). However, several preclinical models of epilepsy have shown varying results regarding changes in the redox mechanism. No changes in the levels of GSH in the cortex were observed at 4 h post-SE, signifying that GSH might play an uneven role in the cortex but not in the hippocampus (Gluck et al., 2000), as few studies reported a reduction in hippocampal redox level after SE (Liang and Patel, 2006; Ong et al., 2000). A time-reliant reduction in GSH/glutathione disulfide (GSSG) ratio along with an adequate surge in glutathione peroxidase (GPx) activity and a reduction in glutathione reductase (GR) activity in tissue homogenates and a mitochondrial fraction of hippocampi, subsequent kainic acid-induced SE, have been documented (Liang and Patel, 2006). Increased neuronal injury in the CA3 region occurs between two to 7 days after KA treatment after the onset of reported redox alterations, proposing that oxidative stress might result in seizure-induced apoptosis (Liang et al., 2000; Patel et al., 2001; Mariani et al., 2005). However, studies of oxidative stress or mitochondrial incapacity in the human brain are scarce due to the small tissue accessibility.

LPO has been extensively used as an oxidative stress marker in experimental animals, observations documented that kainic acid-induced seizure susceptibility is accompanying mitochondrial OS due to elevated mitochondrial LPO and disturbed GSH homeostasis in the hippocampus (Shin et al., 2008). Several human studies have reported a reduction of antioxidant levels in the blood of individuals with myoclonic epilepsy and showed that the Cu–Zn–SOD activity in epileptic patients was lesser than in the control individuals (Ben-Menachem et al., 2000). Similarly, a study reported that the GSH, GPx, total antioxidant status, and vitamin-E levels in erythrocytes of the refractory epilepsy group were lower than in the control (Yürekli and Nazıroğlu, 2013).

NF-E2-related factor 2/antioxidant response element (Nrf2/ARE) signaling pathway is a cytoprotective system that occurs endogenously (Xing et al., 2015). The transcription factor Nrf2 localizes to the cell’s nucleus after activation, forming heterodimers with certain other transcription factors like c-Jun before binding to the ARE (Zhang et al., 2019). Several genes involved in cell anti-inflammatory and antioxidant mechanisms, such as NAD(P)H quinone oxidoreductase 1 (NQO1) and heme oxygenase-1 (HO-1), are regulated by Nrf2-ARE binding (Colín-González et al., 2013; Zhong and Tang, 2016). Furthermore, in animal and cell culture models of neurological illnesses such as Parkinson’s disease, Alzheimer’s disease, and epilepsy, initiation of the Nrf2/ARE pathway plays a crucial role (Calkins et al., 2009; Wang et al., 2014). It has been reported that oxidative stress has a role in the onset and progression of seizures (Sudha et al., 2001). Increased free radicals result in membrane lipid peroxidation and lower glutathione levels in the epileptic brain region (Shi et al., 2018). Furthermore, activation of the Nrf2-ARE pathway in the hippocampus reduced the development of amygdala kindling and improved cognitive impairment and oxidative stress caused by epileptic seizures (Wang et al., 2014; Shi et al., 2015). In conclusion, activating the Nrf2-ARE pathway may significantly protect against seizure-related brain damage.

### 3.4 Epilepsy and cognition impairment

In epilepsy patients, psychiatric comorbidities are relatively prevalent. Cognitive impairment appears to be one of the main comorbidities allied with chronic epilepsy among these comorbidities (Gaitatzis et al., 2004; Kanner, 2016; Zhu et al., 2017). Despite mounting epidemiological and experimental evidence pointing to a link between epilepsy and neuropsychiatric comorbidities such as memory and cognitive deficiency, the molecular processes underlying this association are unknown.

Extended cytokine-mediated inflammatory signaling activation leads to the dysfunction of neurons culminating in cognitive impairments, which have now been understood better (Cunningham and Sanderson, 2008), and increased activation cytokine signaling in the brain hampers memory and learning (Dantzer et al., 2008). Through cytokine-mediated collaborations among neurons and glia, inflammatory processes inside the CNS contribute to cognitive impairment (Wilson et al., 2002). The activated microglia release inflammatory molecules and free radicals, which cause tissue damage and neurotoxicity through processes such as oxidative stress and synaptic reorganization, all of which are linked to cognitive deficits (d’Avila et al., 2018). Memory retention and hippocampal-dependent learning are both facilitated by neurogenesis (Drapeau et al., 2007). Also, neuroinflammation and redox imbalance coupled with mitochondrial stress and dysfunction can negatively impact cognitive performance, either actively or passively, through decreased hippocampal neurogenesis (Kodali et al., 2018). According to growing evidence, cytokines such as IL-1β, IL-6, and TNF-α have been implicated in the molecular mechanisms involved in learning and memory. Synaptic pruning is considered to be induced by TNF-α and IL-1β production, resulting in decreased synaptic plasticity and morphological brain alterations that have a deleterious impact on memory function (Rosenblat et al., 2014). Cytokines appear early during inflammatory events and promote a more lasting inflammatory reaction. These chain of events could be particularly damaging to the susceptible hippocampus area, creating learning and cognitive impairments (Elderkin-Thompson et al., 2012). These inflammatory cytokines’ effects on cognitive functioning can be described as follows: IL-1β may alter neurogenesis and long-term potentiation (LTP); IL-6 influences neural plasticity and neurogenesis; and TNF-α disturbs LTP and synaptic transmission (McAfoose and Baune, 2009). TNF activates two receptors, TNF-p55 (TNFR1) and TNF-p75 (TNFR2), which have a variety of biological consequences. TNF affects synaptic efficiency by increasing surface expression of α-amino-3-hydroxy-5-methyl-4-isoxazolepropionic (AMPA) receptors, which is facilitated by TNFR1 and phosphatidylinositol 3 (PI3) kinase and has the propensity diminish synaptic inhibition via endocytosis of GABA receptor (Stellwagen et al., 2005). TNF’s neuromodulatory effects in the brain, as well as its part in synaptic plasticity, were demonstrated by these molecular actions.

Learning and cognitive decline in epileptic patients is attributed to apoptosis during epileptogenesis (Arend et al., 2018). The extrinsic apoptosis pathway is comprised of insults facilitated by death receptors, while in the intrinsic mitochondrial pathway, death signals can either act in a direct or indirect manner on mitochondria, causing cytochrome C release and ultimately forming apoptosome complex (Kajta, 2004; Krantic et al., 2007). Different expressions of several caspases have been studied in brain samples of epileptic patients; the caspases being confined within soma and dendrites of neuronal cells, displaying caspase-mediated damage of proteins (Schindler et al., 2006; Henshall et al., 2012). In surgically resected neocortex tissues from TLE patients, there was a significant increase in the levels of anti-apoptotic mediators Bcl-2 and Bcl-xL compared to the controls. The Bcl-xL has a direct correlation with the frequency of seizures in an epileptic patient. This signifies that the in the pathogenesis of human epilepsy, both pro-apoptotic and anti-apoptotic mechanisms have contributory roles (Henshall et al., 2000).

Similarly, several other studies have revealed gene expression of Bcl-2, procaspase, and caspase in the hippocampus of patients with TLE (Henshall and Engel, 2013). Also, correlative analysis has shown that apoptosis-related gene expression, including p53, Fas, Bax, Bcl-2, and caspase-3, conferring that apoptotic mechanisms get stimulated in TLE patients with mesial temporal sclerosis (Xu et al., 2007). Furthermore, occasional TUNEL-positive cells with apoptotic cells were present in the hippocampus of TLE patients (Henshall and Engel, 2013).

### 3.5 Epigenetic mechanisms in epilepsy

Epilepsy is a complex disorder that is characterized by alterations in cell functioning which is a cause of complexity. Significant alterations in gene expression and regulation of these gene expressions drive this abnormal cellular function. Recent research has identified epigenetic modulation of gene expression as a significant regulator with rapid and sustained changes. Many elements of cellular physiology are regulated by epigenetic-mediated gene output, including the structure of neurons and release of neurotransmitters, ion channel protein availability, and other essential neuronal activities (Ryley Parrish et al., 2013). Sites for post-translational changes, such as phosphorylation, acetylation, ubiquitination, and methylation, can be found in the N-terminal tails of histone proteins (Davey et al., 2002). Histone alterations can affect the chromatin organisation and subsequent transcriptional activation on their own or in combination with other epigenome features (176,177). Acetylation of lysine residues on histones’ N-terminal tails culminates in a much more transcriptionally favorable situation (Creyghton et al., 2010). Acetylation of histones is regulated by two opposing enzymes histone acetyltransferase (HATs), which is the writer, and the eraser, histone deacetylases (HDACs), which is the eraser (Jagirdar et al., 2015). Acetylation is carried out by HATs, which catalyse the allocation of acetyl moiety to lysine residues, leading to the neutralization of histones’ charge and facilitation of the chromatin structure (Carrozza et al., 2003). HDACs eradicate these acetyl moieties from histones, increase their positive charge, and cause chromatin compaction (Jagirdar et al., 2016). Acetylation of histone is known to have an important homeostatic function in plasticity, and synaptogenesis in the brain (Crosio et al., 2003; Farrelly and Maze, 2019). In both experimental and human TLE, studies have found rapid and permanent modifications in the acetylation of lysine at the promoter region of numerous genes, as well as dissimilarities in the activity of HDAC (Huang et al., 2012; Brennan et al., 2016). Following SE induction, there was a reduction in H4 acetylation at the glutamate receptor, GluR2. In the pilocarpine rat model, however, there was an acetylation upsurge at the brain-derived neurotrophic factor (BDNF) promoter after SE (Huang et al., 2002), and induction of c-fos through epileptic processes implies the role of acetylation in controlling mechanisms involved in epilepsy development (Sng et al., 2006). Also, SE causes energy-dependent HDAC Sirtuin 1 (SIRT1) activation, which further leads to the depletion of H4K16ac, implying a connection between epileptogenic events and epigenetic alteration (Hall et al., 2017). In both preclinical animals of epilepsy and human TLE, HDAC2 expression is increased, which could lead to the deregulation of genes associated with the synaptic function (Huang et al., 2012; Huang et al., 2002). Bromodomains (BrDs) are the proteins involved in reading histone tails (Pal et al., 2003). BRD2 is a member of the BrD subfamily, and it is linked to adolescent-onset epilepsy, known as Juvenile Myoclonic Epilepsy (JME) (Pal et al., 2003). Single nucleotide polymorphisms have been discovered at the promoter region of the BRD2 gene, which could contribute to the progression of JME (Cavalleri et al., 2007).

DNA methylation is the epigenomic change that has received the most attention. The addition of a methyl group covalently from S-adenyl methionine (SAM) to the cytosine at the fifth position generates 5-methylcytosine (5 mC). The enzyme family known as DNA methyltransferases (DNMTs) is responsible for this alteration. DNMTs are classified into three families: DNM3b, DNMT1, and DNMT3a (Fatemi et al., 2005; Kinde et al., 2015). DNMT3a and DNMT3b causes the methylation of novel sites on the genome, while DNMT1 keeps pre-existing methylation marks (Song et al., 2012). Depending on the methylation site and the conditions involved, DNA methylation can both stimulate and suppress gene expression (Barrès et al., 2009) even though it is most commonly associated with gene repression (Iguchi-Ariga and Schaffner, 1989; Bird and Wolffe, 1999). The predominance of DNA methylation occurs at CpG dinucleotide and is linked to transcriptional inhibition (Fatemi et al., 2005). CpG islands have a high frequency of promoter regions, which improve availability and stimulate transcription, but methylation of CpG has the reverse effect (Jeziorska et al., 2017). DNA methylation is now known to be dynamic, with methyl groups that can be modified or erased from DNA by eraser enzymes (Ma et al., 2009). DNA methylation is expected in the nervous system and is required for appropriate brain function, especially development and neuronal plasticity (Tsankova et al., 2007; Borrelli et al., 2008; Feng et al., 2010). A variety of illness conditions, including a number of epileptic syndromes, have been associated with abnormal methylation patterns (Kobow et al., 2009; Miller-Delaney et al., 2012; Dębski et al., 2016). Kobow and co-workers demonstrated changes in DNA methylation in rats with chronic epilepsy and hypothesised that DNA methylation might play a pathomechanistic function in the process of epileptogenesis, thus contributing to gene dysregulation (Kobow et al., 2013). Hypermethylation was found to be prevalent in the pilocarpine model of TLE in rats, and it was linked to downregulated gene expression (Kobow et al., 2013). Further research has discovered that epilepsy is caused by a change in the levels of DNA modifying enzymes, namely, DNMT1 and DNMT3a, reportedly elevated in TLE, mainly in neural cells, implying that *de novo* methylation and its regulation has a role to play in human TLE (Zhu et al., 2012). William-Karnesky and colleagues provided important functional insights by showing that inhibiting methylation of DNA in different experimental models of seizures could reduce the genesis of epilepsy. The researchers utilised adenosine increase to reverse the DNA hypermethylation and discovered that it stopped mossy fibre sprouting in the region of the hippocampus and stopped the progression of the disease for 3 months. The relevance of aberrant DNA methylation in the process of epileptogenesis was underlined in this work, with reversal slowing disease development (Williams-Karnesky et al., 2013). Intriguingly, activation of the focal amygdala led to reduced promoter methylation and enhanced gene expression (Dębski et al., 2016). Dębski et al. (2016), experimented with DNA methylation profiles in epilepsy animal models such as pilocarpine, traumatic brain injury, and amygdala stimulation and reported no resemblance in differentially methylated genes. These findings suggest that methylation of DNA is very context-dependent, and epilepsy’s aetiology has a significant impact on genetic expression and epigenetic alterations (Dębski et al., 2016). As there were diverse methodologies utilized to collect samples from models of seizures used, this may be possible that factors like anesthetic regimes, rodent strain, etc., influenced the observed methylation patterns (Dębski et al., 2016). In the intra-amygdala KA model of TLE in mice, a genome-wide methylation investigation found substantial differential methylation compared with controls, with hypermethylation being the maximum evident (Miller-Delaney et al., 2012). In human TLE, 146 genes presented changed DNA methylation patterns with hippocampal sclerosis, most of which were hypermethylated. Furthermore, methylation at the promoter regions and start sites of transcription was found to be responsive to several microRNA-encoding genes (Miller-Delaney et al., 2015).

## 4 Overview of curcumin's effect on epilepsy

### 4.1 Effect of curcumin on inflammatory mediators

According to a growing body of research, epileptogenesis is supposed to be aided by cellular inflammatory signaling in the brain. Based on accumulated evidence from clinical and preclinical studies, seizure causes the release of inflammatory mediators at the cellular and molecular level, which has the potential to cause brain excitability and neuronal degeneration (Vezzani et al., 2011). Both clinical and experimental acquired epilepsies demonstrated relationship between seizures and inflammatory mediators (Friedman and Dingledine, 2011; de Vries et al., 2012; Devinsky et al., 2013; Aronica et al., 2017), whereas the involvement of inflammation in hereditary epilepsies is a relatively recent area of study for scientists (Shandra et al., 2017).

In surgically isolated brain tissues from drug-resistant epilepsy patients showed the presence of the inflammatory cascade from drug-resistant epilepsy patients, with reactive gliosis and upregulation of chemokines and cytokines. Chemical or electrical seizures in experimental models of epilepsy cause fast growth of an inflammatory process in the brain (Vezzani et al., 2013) and other areas of the CNS. Glial cells rapidly release cytokines such as IL-1β, TNF-α, IL-6, and HMGB1 (Vezzani et al., 2011), and the expression of cytokine receptors is increased in neurons, microglia, and astrocytes (Balosso et al., 2005) after seizures. In addition to cytokine production, other types of inflammatory mediators, such as prostaglandins (PG), significantly rise after seizures (Shimada et al., 2014). Following seizures, the enzyme cyclooxygenase-2 (COX-2) is rapidly activated in the brain, which is responsible for PG production (Yoshikawa et al., 2006). Experimental and human TLE are both associated with a substantial activation of the classical complement pathway (Aronica et al., 2007). During epileptic episodes, chemokines and associated receptors are also produced (Foresti et al., 2009; Cerri et al., 2016). Cytokines interact with their receptors and activate signaling pathways that cause the production of chemokines, cytokines, enzymes, and receptors, resulting in an inflammatory response. For example, IL-1β and HMGB1 bind to IL-1R1 and TLR-4, respectively, activating intracellular pathways that lead to the NF-κB. The transcription factor NF-κB regulates the expression of several genes involved in neuroinflammation, synaptic plasticity, and cell death/survival and is a common molecule triggered by inflammatory ligands (O’Neill and Kaltschmidt, 1997). As evidenced by the current literature, neuroinflammatory pathways play a critical role in seizure initiation and progression (Balosso et al., 2005; Maroso et al., 2010). By mediating increased calcium influx through NMDA receptors, IL-1β, for example, exerts powerful pro-convulsant effects (Vezzani et al., 2013). Chemokines directly impact neuronal excitability by interacting with receptors expressed at both the presynaptic and postsynaptic levels (Rostène et al., 2007). In cerebellar neurons, for example, CCL2 can modify electrophysiological properties and calcium signaling (van Gassen et al., 2005), inhibits inhibitory responses in neurons in the spinal cord (Gosselin et al., 2005), and in the Schaffer collateral circuit of the hippocampus, potentiates excitatory postsynaptic currents (Zhou et al., 2011). The p38 MAP kinase pathway is critical for mediating the effects of chemokines (Cho and Gruol, 2008). Chemokines are overexpressed in epileptic patients, according to several studies. CCL2 has also been discovered to be substantially expressed in surgically removed brain tissues (Choi et al., 2009), and CCL3 and CCL4 levels have been found to be elevated in MTLE patients (van Gassen et al., 2008). Furthermore, in MTLE models, the CC chemokine receptor 5 (CCR5) is upregulated in the brain vasculature (Louboutin and Strayer, 2013).

**FIGURE 2:**
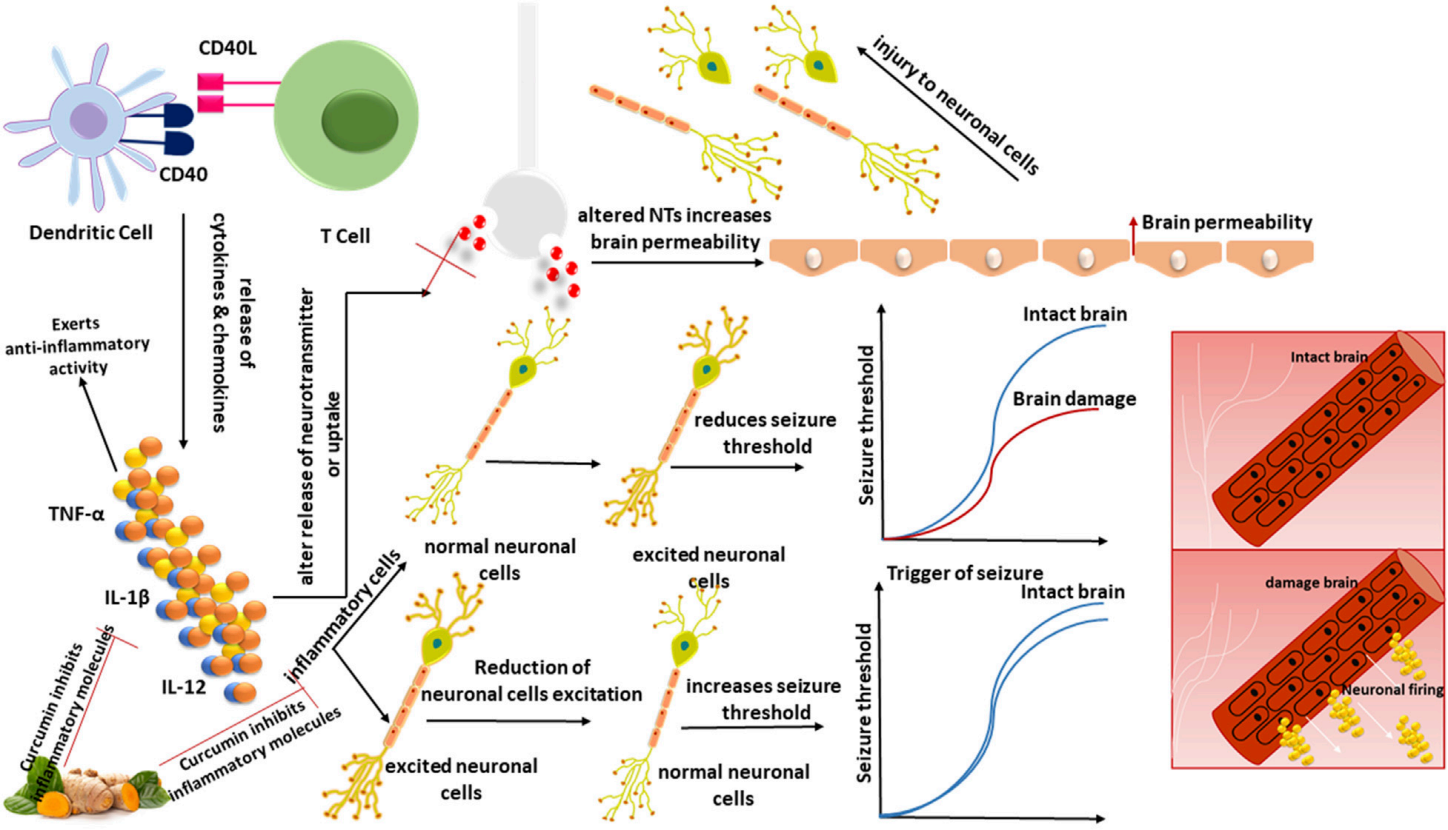
TNF-α, IL-1β, and IL-12 are among the cytokines and chemokines produced due to the interaction between DCs and T-cells via CD40:CD40L. Inflammatory cells raise BBB permeability, modify neurotransmitter release or uptake, damage neuronal cells, and secrete cytokines, raising neuronal excitement and lowering the seizure threshold. Reducing inflammatory mediators in the brain regions demonstrates curcumin’s anti-inflammatory effect.

According to studies, curcumin can modify these inflammatory pathways linked to neurological diseases. Hyperactivity of cyclin-dependent kinase 5 (Cdk5)/p25 is linked to the production of amyloid and tau pathology in Alzheimer’s disease (AD). Curcumin inhibited p25-mediated glial activation and the generation of pro-inflammatory chemokines/cytokines in an animal model (Sundaram et al., 2017). Curcumin’s anti-inflammatory activity was further shown in a recent study by reducing TNF-α and IL-6 in the brain areas of a middle cerebral artery blockage stroke model (Zhang et al., 2017). Curcumin has been shown to suppress astrocyte activation by inhibiting the NF-κB signaling pathway, which resulted in decreased astrocyte release of the chemokines MCP-1, RANTES, and CXCL10, as well as decreased macrophage and T-cell infiltration, reducing inflammation in the glial scar in the spinal cord injury (Yuan et al., 2017). Curcumin inhibited the expression of T-cell co-stimulatory molecules (CD80 and CD86) and MHC class II, decreased the levels of pro-inflammatory cytokines (IL-17, IFN-γ, and TNF-α), increased the levels of anti-inflammatory cytokine IL-10, and increased the number of NKR-P1 (+) cells (natural killer cell receptor protein 1 positive cells) in an animal model of myasthenia gravis (Wang et al., 2016). Huang et al. (2018), have discovered that curcumin protects neurons by inhibiting autophagic activity via the PI3K/Akt/mTOR pathway, as well as suppressing inflammatory mediators via the TLR4/p38/MAPK signaling. Curcumin was reported to be efficient in reducing glial activation in the PTZ kindling epilepsy model. In the brains of kindled rodents, mRNA and protein levels of pro-inflammatory cytokines (IL-1β, IL-6, TNF-α), and chemokines (MCP-1) were elevated, which was decreased by curcumin administration (Kaur et al., 2015) (Figure 2). The effect of curcumin on inflammatory mediators induced during epilepsy, is shown in Table 1.

**TABLE 1:** Effect of curcumin administration on neuroinflammation induced during epilepsy.

S.No.	Experimental model of epilepsy	Species used	Dose of curcumin	Route of administration	Duration of intervention	Findings	Reference
1	KA-induced SE	Wistar rats	100 mg/kg	Intraperitoneal	14 days	Attenuation of astrocytes activation in the hippocampus, reduction of IL-1β and TNF-α levels in the hippocampus	Jiang et al. (2015)
2	PTZ-induced kindling	Wistar rats	100 mg/kg	Oral	30 days	Significant lowering of cytokines (IL-6, IL-1β, TNF-α) and chemokine (MCP-1) activation in kindled rats (in both hippocampus and cortex). Upregulated expression of Iba-1 and GFAP in curcumin-treated experimental animals. Moreover, a significant lowering of activated glial cell number was observed upon administration of curcumin	Kaur et al. (2015)
3	Organotypic hippocampal-entorhinal cortex slice culture	Sprague Dawley rats	Concentration in medium: 0.05%	NA	21 days	Lowering of pS6 expression at one phosphorylation site. Downregulation of mRNA expression of IL‐1β, IL‐6 and TGF-β	Drion et al. (2019)
4	KA-induced epilepsy	Sprague Dawley rats	100 mg/kg	Oral	7 days	IL-1β and NLRP3 expression were reduced upon curcumin administration. Curcumin repressed the NLPR3/inflammasome activation and decreased neuronal loss in the hippocampus region	He et al. (2018)
5	*In vitro*: Astrocyte cell culture *In vivo*: electrically induced SE	Human fetal brain tissue, Sprague Dolly rats	*In vitro*: 10 μM, *In vivo*: 2 μL of 2 mM curcumin solution	Intracerebral ventricle	7 days	Curcumin suppressed the MAPK pathway in cultured astrocytes; curcumin decreased the expression of IL-6 and COX-2 in cultured astrocytes. In electrically induced SE, curcumin administration did not suppress the inflammatory markers	Drion et al. (2018)

**Abbreviations: KA**, Kainic acid; **SE**, Status epilepticus; **IL-1β**, Interleukin-1 beta; **TNF-α,** Tumour necrosis factor-alpha; **IL-6**, Interleukin-6; **MCP-1**, Monocyte chemoattractant protein -1; **GFAP**, Glial fibrillary ancillary protein; **TGF-β**, transforming growth factor-β; **NLRP3,** NLR family pyrin domain containing 3; **SE**, Status epilepticus; **MAPK**, mitogen-activated protein kinase; **COX-2**, Cyclooxygenase-2.

### 4.2 Effect of curcumin on dendritic cells

Curcumin suppresses the maturation and activity of murine bone marrow-derived DCs (BMDCs) by lowering the expression of CD86, CD80, and MHC-II molecules (Kim et al., 2005). The secretion of IL-12 and other inflammatory cytokines was found to be decreased in curcumin-treated BMDCs, resulting in a suppression of Th1-mediated immune responses. Curcumin was discovered to retain DCs in an immature stage in addition to its inhibitory effects on BMDCs (Kim et al., 2005). The effects of curcumin and hydroethanolic extracts of turmeric were tested on human DCs in *in-vitro* conditions. Decreased expression of co-stimulatory molecules (CD86, CD80, and CD83), lower production of cytokines, as well as reduction of CD4^+^ proliferation were all discovered in the study (Krasovsky et al., 2009; Shirley et al., 2008). Additionally, when DCs were treated with curcumin, both chemokine release and cell movement were inhibited, resulting in less communication between DCs and T-cells (Shirley et al., 2008). Curcumin-treated DCs have phenotypic characteristics that are comparable to iDCs, and T-cells interacting with these DCs possess tolerogenic functions (Shirley et al., 2008). Curcumin has been demonstrated to reduce the expression of MHC-II molecules, co-stimulatory molecules (CD80, CD40, CD86, CD252, CD205, CD54, and CD256), and inflammatory cytokines in CD8^+^ DCs, hence slowing their maturation and eventual Th1 response (Zhao et al., 2016a; Zhao et al., 2016b). The iDCs were discovered to be incapable of competently triggering the T-cell responses, resulting in an upsurge in the frequency of Tregs in gut-associated lymphoid tissue, providing a “recovery effect” (Zhao et al., 2016a). Similarly, it has been observed that injecting curcumin-treated DCs in BALB/c mice causes CD4^+^CD25+FoxP3+ Treg cells to proliferate (Rogers et al., 2010). Curcumin inhibited STAT1 activation in bone marrow-derived DCs by impeding Janus-activated kinase 1/2 and protein kinase C delta phosphorylation, preventing STAT1 translocation and attaching to the GAS component of the IRF-1 promoter (Jeong et al., 2009). Curcumin has immunomodulatory effects on the AP1 and NF-κB pathways (activator protein) pathways (Ruzicka et al., 2018). Curcumin can also inhibit DC stimulation and maturation by modulating the Janus Kinase (JAK)/STAT pathway (Zhao et al., 2016b). Curcumin derivatives have also been shown to inhibit NF-κB and MAPK pathways by downregulating the downstream targets of the IKK/IκB/NF-κB and c-Raf/MEK/ERK inflammatory cascades (Razali et al., 2018). Curcumin alleviated asthma symptoms in a mouse model by stimulating the Wnt/β-catenin signaling pathway in DCs, which caused decreased expression of co-stimulatory markers (CD86, CD40, and CD11c), as well as decreased CD4^+^ T-cells activation and proliferation (Yang et al., 2017). Curcumin, owing to its anti-inflammatory activities, appears to alter several immune-mediated responses, and so can be a probable therapeutic compound for the management and treatment of seizures, according to accumulating experimental evidence.

### 4.3 Effect of curcumin on oxidative stress

Curcumin appeared to provide neuroprotection against oxidative stress, according to a growing body of research, by modulating oxidative stress-induced brain injury and a cascade of inflammation and apoptotic signaling (Tiwari and Chopra, 2012). Curcumin increases cell survival in the hippocampus by reducing oxidative stress, as seen by reduced malondialdehyde and glutathione levels (Keskin-Aktan et al., 2018).

**FIGURE 3:**
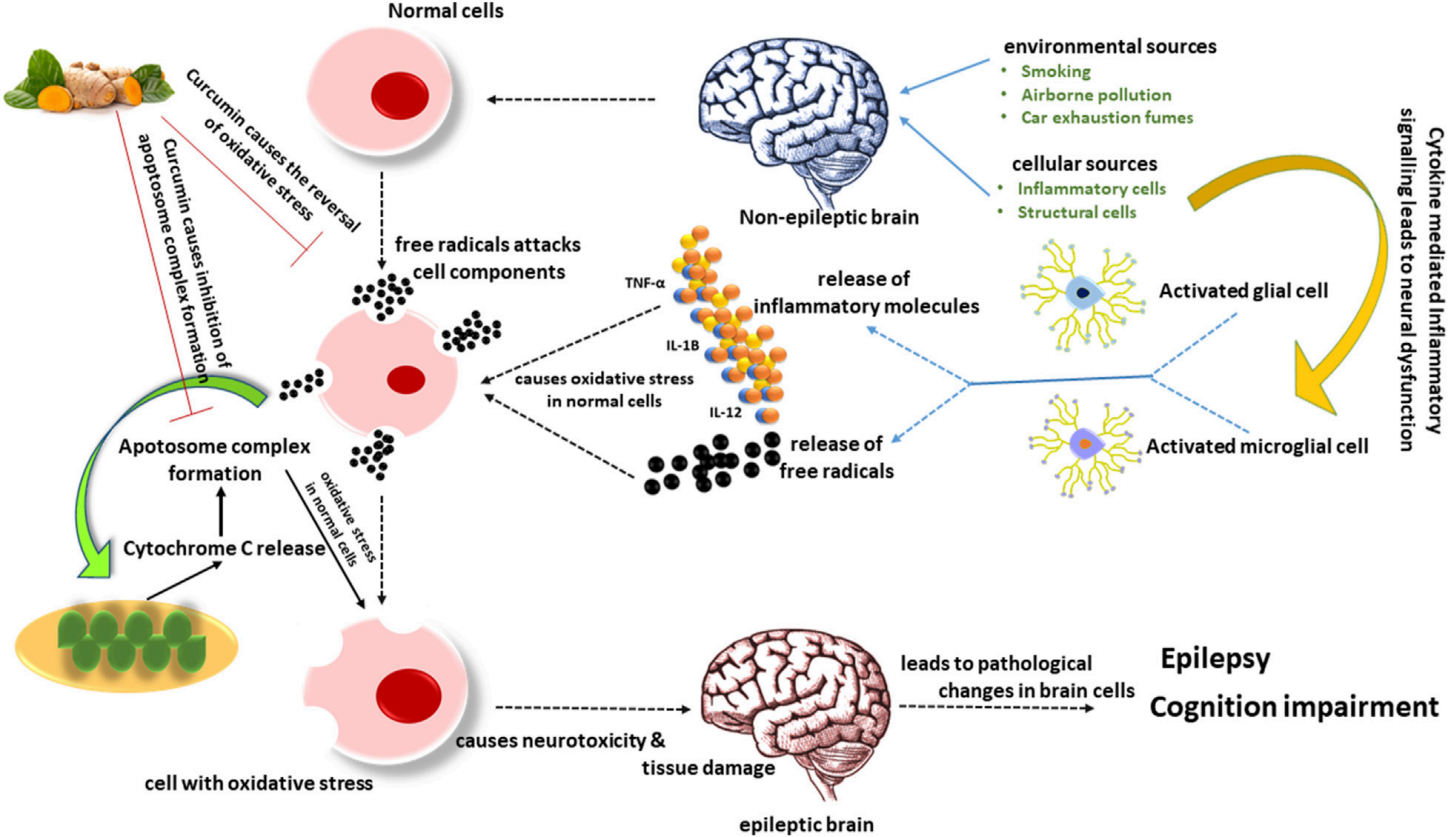
Activated microglia generate inflammatory chemicals and free radicals that damage tissue and cause neurotoxicity through synaptic rearrangement, oxidative stress, and other mechanisms associated with cognitive problems. The extrinsic pathway for apoptosis is composed of insults that death receptors facilitate. In contrast, the intrinsic mitochondrial pathway is composed of death signals that can act directly or indirectly on mitochondria, causing the release of cytochrome C and ultimately forming an apoptosome complex. The existing evidence unequivocally demonstrates that neuronal hyperactivity is associated with increased free radical generation, notably in brain tissue. According to reports, curcumin prevents the production of free radicals.

Nrf2 is a widely distributed protein in the central nervous system that has been considered a significant regulator of brain inflammation and oxidative stress. Curcumin enone compounds can activate Nrf2, a transcription factor that regulates phase II detoxification and antioxidant genes (Deck et al., 2018). Benzodiazepines (BZDs) are a class of medications used as anxiolytics, sedatives, and, most significantly, anticonvulsants (Griffin et al., 2013). They are more recognized for clinical concerns like cognition and neuropsychomotor deficiency and their role in long-term memory impairment (Buffett-Jerrott and Stewart, 2002). Curcumin reduced the levels of oxidative stress in the blood and the hippocampus and decreased the extracellular signal-regulated kinase (ERK 1/2)/nuclear transcription factor-NF-κB/pNF-κB pathway in a diazepam-induced cognition impairment model, according to a recent study (Sevastre-Berghian et al., 2017). Curcumin can also prevent early brain injury by reducing oxidative stress caused by subarachnoid hemorrhage by inhibiting NF-κB activation (Cai et al., 2017). With the systemic injection of kainate, curcumin can prevent hippocampus cell loss and lessen seizures in mice (Shin et al., 2007). In a kainic acid epilepsy model, curcumin drastically reduced MDA levels and improved glutathione levels, which are oxidative stress markers (Gupta et al., 2009). Curcumin pre-treatment reduced oxidative stress indicators in a comparable model in another investigation (Kiasalari et al., 2013). PTZ-kindling is a type of persistent epilepsy marked by a steady rise in seizure susceptibility. PTZ kindling promotes molecular and cellular changes in the hippocampus, which are accountable for generated free radicals and neurodegeneration (Zhu et al., 2017). In the rat brain, PTZ kindling raised MDA levels and decreased GSH levels, indicating cellular oxidative stress, while curcumin reduced both (Mehla et al., 2010). In PTZ rodents supplemented with curcumin, considerable antioxidant activity was obvious from levels of lipid peroxidation and protein carbonyls, which is consistent with earlier research (Kaur et al., 2014). In one of the most ground-breaking studies, it was discovered that combining curcumin with well-known AEDs like phenobarbitone, phenytoin, and carbamazepine in sub-therapeutic doses prevented learning and memory impairment caused by seizures, whereas sub-therapeutic administered doses of AEDs alone had no effect. Curcumin also reduces the oxidative imbalance caused by seizures (Reeta et al., 2011). The iron-induced epilepsy model is a well-known human posttraumatic epilepsy model (Willmore et al., 1978). Free iron in the brain causes cellular-thiol processes to be disrupted, as well as the formation of free radicals generated. Curcumin reduced protein oxidation by a large amount, which may be due to its capacity to pass the blood–brain barrier (Wang et al., 2014) and bind with freely available Fe^2+^/Fe^3+^ ions (Baum and Ng, 2004). In epileptic models, membrane fluidity is impeded, and curcumin’s antioxidant properties may help to reduce the damage. Curcumin also inhibits PKC-mediated excitotoxicity by preventing oxidative modification of the protein kinase C (PKC) regulatory component, which restricts the activation of cytosol PKC activity (Jyoti et al., 2009). SE is a neurological disorder in which recurrent generalized convulsions last more than 30 min and result in significant neuronal damage. The available research clearly shows that neuronal hyperactivity has been linked to increased production of free radicals, particularly in brain tissue (Freitas et al., 2005). In the hippocampus and striatum parts of the brain, a study found a large increase in lipid peroxidation and a considerable drop in GSH, indicating oxidative stress, in agreement with previous research. Curcumin pre-treatment reduced the disruption of oxidative stress-related enzymes in a dose-dependent way (Ahmad, 2013) (Figure 3). The effect of curcumin on oxidative stress, represents in Table 2.

**TABLE 2:** Effect of curcumin administration on oxidative stress associated with seizures.

S.No.	Experimental model of epilepsy	Species used	Dose of curcumin	Route of administration	Duration of intervention	Findings	Reference
1	PTZ and MES- induced seizures	Wistar rats	Curcumin in the dose 300 mg/kg co-administered with phenytoin, valproate, phenobarbitone and carbamazepine (sub-therapeutic dose)	Oral	Once	Valproate and curcumin + valproate treatments significantly decreased the level of MDA and increased the GSH level in the whole brain tissue when compared with the disease group	Reeta et al. (2011)
2	PTZ-induced kindling	Wistar rats	100, 200 and 300 mg/kg	Oral	35 days	Curcumin administration reduced MDA levels and increased GSH levels in the whole brain dose-dependently	Mehla et al. (2010)
3	PTZ-induced kindling	BALB/c nude mice	60 mg/kg	NA	NA	Curcumin administration led to the upregulation of cysteine, which is a critical reductive biothiol, and oxidative stress in the brain	Li et al. (2020)
4	PTZ-induced kindling	Wistar rats	100 mg/kg	Oral	30 days	Mitochondrial swelling was reduced (by two folds), increased cytochrome c oxidase activity, protein carbonyl levels relatively increased, and restoration of GSH levels in the hippocampus and cortex. Reversal of morphological deficits of mitochondria, restoration of neuronal loss	Kaur et al. (2014)
5	Kainate-induced model of TLE	Wistar rats	100 mg/kg	Oral	7 days	Reduction in levels of MDA, nitrite, and nitrates. Amelioration of neuronal damage in CA1, CA3, and hilar regions, reduction of MFS	Kiasalari et al. (2013)
6	Lithium-pilocarpine model of SE	Sprague–Dawley rats	50, 100 and 200 mg/kg	Oral	3 days	Reduction in levels of TBARS, dose-dependently in the hippocampus and striatum, increase in levels of GSH in the hippocampus and striatum	Ahmad (2013)

**Abbreviations: PTZ,** Pentylenetetrazole; **MES,** Maximal electroshock seizures; **MDA,** Malondialdehyde; **GSH,** Glutathione; **TLE,** Temporal lobe epilepsy; **MFS,** Mossy fiber sprouting; **CA,** Cornu Ammonis; **TBARS,** Thiobarbituric acid reactive substances.

### 4.4 Effect of curcumin on cognition impairment

The hippocampus region is important for memory and is frequently the site of epileptic episodes. As one of the maximum electrically excited regions of the brain, hippocampal sclerosis is the most commonly diagnosed substrate in mesial temporal lobe epilepsy in both kindling models and TLE patients (Altintaş et al., 2017; Becker, 2018). Epileptic seizures cause neurodegenerative alterations, leading to cognitive impairment (Tai et al., 2018). Long-term TLE is well-documented, with cognitive impairment as a comorbidity (Allone et al., 2017; Rzezak et al., 2017). Multiple epileptogenic and neurogenic alterations also play a role in the evolution of status epilepticus-induced damage and learning and memory problems (Castro et al., 2017). However, current research suggests that neurotransmitter imbalance and changes in neuronal structure due to seizures may play a key role in neurobehavioral changes (Ahmad, 2013; Kubová et al., 2004). Patients with epilepsy frequently experience cognitive deficits (Rodríguez-Cruces et al., 2018). Furthermore, the parahippocampal cortex is linked to memory impairment in patients with mesial TLE and hippocampal sclerosis (Colnaghi et al., 2017). Nearly 30% of epilepsy patients treated with AEDs experience cognitive impairment as a side effect (Hernández et al., 2005; Dalic and Cook, 2016); with higher doses of AEDs, the risk of cognitive impairment increases (Jokeit et al., 2005).

In the kindling model of epilepsy, polyphenolic substances like epigallocatechin gallate are beneficial as a prophylactic agent for cognition impairment (Xie et al., 2012). In a Parkinson’s disease mouse model, nanoparticles containing curcumin and piperine dramatically restored the neurobehavioral impairments and neuronal degeneration caused in the substantia nigra (Kundu et al., 2016). In mice, functionalized polymerosomes containing curcumin reached the brain and reversed cognitive impairment caused by amyloid-β_1-42_ (Jia et al., 2016). Furthermore, demethoxycurcumin, a natural derivative of curcumin, can alleviate memory impairments caused by scopolamine treatment via changing hippocampus choline acetyltransferase expression (Lim et al., 2016). In cadmium-induced memory impairment, inhibition of AChE and adenosine deaminase activities, as well as an increase in the antioxidant quality of curcumin, provided improved cognitive qualities (Akinyemi et al., 2017). Curcumin administration has been proven to improve cognitive skills by lowering lipid peroxidation in the brain tissue of elderly female rats, according to a study (Belviranlı et al., 2013). Other studies also found curcumin reverses cognitive impairment dose-dependently (Mehla et al., 2010; Jyoti et al., 2009). Another study supports the neuroprotective efficacy of curcumin, where curcumin administration significantly attenuated cognitive decline associated with the experimental model of chronic epilepsy (Kaur et al., 2015). Reeta et al. (2011) found that when curcumin was combined with subtherapeutic doses of AEDs such as valproate, phenobarbitone, phenytoin, and carbamazepine, cognitive abilities were improved. Because of its potential to alter the central monoaminergic cascade, curcumin ameliorates depression-like behavior and memory loss in PTZ-treated mice (Choudhary et al., 2013). In non-demented persons, a highly absorbable curcumin formulation showed improvement in the memory and attention. Curcumin appears to be an adjuvant therapy for older people with AD and mild cognitive impairment, preventing progressive loss of cognitive abilities by stabilizing the disease course (Dost et al., 2021). As discussed, curcumin has inhibitory potential on inflammatory mediators, such as NF-κB and COX-2, involved in the controlling expression of anti-apoptotic genes like Bcl-2 (Mortezaee et al., 2019).

A study reported curcumin alleviates cerebral hypoperfusion-induced cognitive impairment in rats through suppression of apoptosis (Zheng et al., 2021). In another study, curcumin stimulated the AMPK-JNK pathway, which caused inhibition of mTOR and upregulation of Bcl-2, subsequently enhancing autophagy, suppressing apoptosis, and enhancing cognitive behavior (Yi et al., 2020). Curcumin encapsulated in solid lipid nanoparticles (SLNs) showed better anti-epileptic efficacy and behavior performance in mice (Huang et al., 2020). Interestingly, a study demonstrated curcumin supplementation in rats treated with gentamicin and sodium salicylates reduced caspase-3 levels, which is the pathological mechanism responsible for its improved locomotor and memory function (Abd-Elhakim et al., 2021). Xiang et al. (2021) reported that curcumin treatment in copper-induced neurotoxicity lessened the reduction in mitochondrial membrane potential and translocation of cytochrome c to the nuclear region. The levels of procaspase 3 and procaspase 9 were upregulated upon curcumin administration. In the colistin-induced peripheral neurotoxicity model of mice, curcumin supplementation at 200 mg/kg body weight downregulated the mRNA expression of Bax, caspase-3, and caspase-9. Subsequent decreased levels of caspase-3 and caspase-9. Table 3 lists the cognition-enhancing property of curcumin in epilepsy.

**TABLE 3:** Effect of curcumin administration on cognition impairment associated with epilepsy.

S.No.	Experimental model of epilepsy	Species used	Dose of curcumin	Route of administration	Duration of intervention	Findings	Reference
1	PTZ-induced kindling	Wistar rats	100 mg/kg	Oral	30 days	Decreased escape latency in MWM suggestive of improved cognitive functions	Kaur et al. (2015)
2	KA-induced status epilepticus	Wistar rats	100 mg/kg	Intraperitoneal	14 days	Decreased neuronal loss in DH and CA3 region, reduced severity of MFS, a significant reduction in escape latency in MWM	Jiang et al. (2015)
3	PTZ-induced kindling	Swiss Albino mice	50, 100 and 200 mg/kg	Intraperitoneal	15 days	Ameliorative effect on depressive behavior and cognitive dysfunction, monoaminergic modulation, inhibition of nitrosative stress, and acetylcholinesterase activity	Choudhary et al. (2013)
4	PTZ and MES- induced seizures	Wistar rats	Curcumin (300 mg/kg) co-administered with phenytoin, valproate, phenobarbitone and carbamazepine (sub-therapeutic dose)	Oral	1 day	Curcumin co-administration with clinically approved AEDs in sub-therapeutic levels avoided learning and memory deficits	Reeta et al. (2011)
5	PTZ-induced kindling	Wistar rats	100, 200, and 300 mg/kg, orally	Oral	35 days	Cognitive impairment ameliorated after curcumin pre-treatment in a dose-dependent manner	Mehla et al. (2010)
6	KA-induced epilepsy	Sprague Dawley rats	100 mg/kg	Oral	7 days	Improvement of recognition efficiency	He et al. (2018)
7	PTZ-induced kindling	Wistar rats	100 mg/kg	Oral	30 days	Curcumin-treated rats performed better in consolidated and long-term memory assessment	Kaur et al. (2014)

**Abbreviations: PTZ,** Pentylenetetrazole; **MWM,** Morris water maze; **KA,** Kainic acid; **DH,** Dentate hilus; **CA3,** Cornu Ammonis; **MFS,** Mossy fiber sprouting; **MES,** Maximal electroshock seizures; **AEDs,** Anti-epileptic drugs.

### 4.5 Effect of curcumin on epigenetic mechanisms

The HDACs inhibition using various substances is considered an epilepsy therapeutic approach because of their ability to regulate several cellular processes (Citraro et al., 2017; Reddy et al., 2018). Curcumin has been reported to be the most powerful inhibitor of HDACs/HATs due to its potential impact on their activity (Hassan et al., 2019). Curcumin has also been shown to be more effective than valproic acid and sodium butyrate, which are established HDAC inhibitors. Also, curcumin drastically lowered the levels of class I HDACs, increasing acetylation (Liu et al., 2005; Chen et al., 2007).

It is reported that curcumin increased SOCS1 and SOCS3 expression, which are suppressors of cytokine signaling, by initiating histone acetylation in SOCS1 and SOCS3 promoter regions in HEL cells. As an inhibitor of HDACs, curcumin suppressed the HDAC enzyme activity and reduced the HDAC1, 3, and 8 levels. Further, curcumin efficiently inhibited HDAC activity and lowered levels of HDAC8 in primary myeloproliferative neoplasms cells (Chen et al., 2013). A study reported that intranasal administration of curcumin inhibited asthma symptoms by disturbing HDAC1 activity leading to NF-κB inhibition in a rodent model of allergic asthma (Islam et al., 2022). Also, curcumin treatment showed elevated expression of acetylated histone H4 and downregulated expression of class I HDAC in the human B lymphoblastoid cell line (Liu et al., 2005). Inhibition of HDAC4s transcription, causing reduction of HDAC expression, has been shown with curcumin treatment in the medulloblastoma cells (Lee et al., 2011). These studies have proposed curcumin as a strong suppressor of HDAC activity.

Curcumin’s effects on DNA methylation have been reported in numerous studies. Curcumin has been demonstrated to suppress the action of DNMTs, resulting in significant changes in DNA methylation patterns in various tumour cells (Liu et al., 2009; Parashar et al., 2012). As per molecular docking studies, curcumin covalently inhibits the catalytic thiolate of DNMT1, culminating in hypomethylation (Liu et al., 2009). In global DNA methylation experiments, curcumin caused reversed DNA methylation in Leukemia cells, similar to decitabine (a powerful hypo-methylating drug). It also caused demethylation and Neurog1 expression in LNCaP prostate cancer cells (Shu et al., 2011). Curcumin did not affect global hypo-methylation of DNA in human colon cancer cells, and it did not change the methylation pattern of long interspersed nuclear elements-1. However, it diminished the methylation of genes involved in the NF-κB pathway. Curcumin’s hypo-methylating effect was found to be correlated with the intensity of methylation because of the selective demethylation of partially methylated CpG sites rather than completely methylated genes (Link et al., 2013). Furthermore, curcumin administration resulted in the reactivation of silent tumour suppressor genes by causing the demethylation of their promoters, resulting in significant tumour suppression (Jha et al., 2010; Yu et al., 2013). It also caused the methylation of the Nrf2 promoter to be reversed in prostate cancer cells (Khor et al., 2011).

## 5 Newer delivery systems for enhanced curcumin’s bioavailability

A nanocarrier system is a drug delivery system that uses tiny particles (nanoparticles) to transport drugs to targeted sites in the body. These particles can be made from various materials, including lipids, polymers, and metals. The nanocarriers can protect the drugs from degradation in the body, increase their solubility, and provide controlled release, enhancing their therapeutic efficacy and reducing potential side effects. Different types of nanocarrier systems for drug delivery exist, such as liposomes, polymeric nanoparticles, dendrimers, and metallic nanoparticles. Liposomes are spherical vesicles made of lipid bilayers that can encapsulate hydrophobic and hydrophilic drugs. Polymeric nanoparticles are biocompatible polymers that can entrap drugs within the particles or conjugate the drugs to the particle surface. Dendrimers are highly branched, spherical molecules that can encapsulate drugs within their cavities or conjugate drugs to their surface. Metallic nanoparticles, such as gold and silver, can also be used for drug delivery and imaging.

Nanocarrier systems have several advantages over conventional drug delivery methods, including improved drug bioavailability and efficacy, reduced toxicity, targeted drug delivery, and prolonged drug release. However, there are also some challenges associated with the development and use of nanocarrier systems, such as their potential toxicity, stability, and manufacturing scalability. Curcumin’s therapeutic applicability is hampered by its poor solubility, physicochemical instabilities, less bioavailability, quick metabolism, and poor pharmacokinetics, despite its vast neuroprotective and disease-modifying properties (308). However, these issues can be addressed by inventing effective delivery systems, and the nanonization concept has offered a platform for overcoming all the barriers to effective curcumin delivery (309). Overall, nanocarrier systems have the potential to revolutionize drug delivery by enabling more effective and targeted therapies for a range of diseases, including cancer, cardiovascular disease, and neurological disorders. There are several different types of nanocarrier systems for drug delivery, each with its unique properties and advantages. Following are the examples of nanocarrier systems.

### 5.1 Solid nanoparticles

One approach to improve the therapeutic efficacy of curcumin is to formulate it as solid nanoparticles. Solid nanoparticles can enhance the solubility and bioavailability of curcumin, as well as provide sustained release, targeted delivery, and protection from degradation (Khairnar et al., 2022). There are several methods for preparing curcumin solid nanoparticles, including (i) Precipitation method: Curcumin is dissolved in an organic solvent and then rapidly added to an aqueous solution containing a surfactant. The resulting nanoparticle suspension is then dried and collected. (ii) Emulsion solvent evaporation method: Curcumin is dissolved in an organic solvent, and then the organic phase is emulsified in an aqueous solution containing a surfactant. The organic solvent is then evaporated, resulting in the formation of solid curcumin nanoparticles. (iii) Supercritical fluid technology: Curcumin is dissolved in a supercritical fluid (e.g., carbon dioxide), and then the supercritical fluid is rapidly expanded to form nanoparticles. (iv) Antisolvent precipitation method: Curcumin is dissolved in a solvent, and then an antisolvent (e.g., water) is rapidly added to the solution, causing the curcumin to precipitate and form nanoparticles.

### 5.2 Biodegradable lipid nanoparticles

BLNPs are a type of drug delivery system that can be used to encapsulate and deliver therapeutic agents, such as drugs or nucleic acids, to target cells or tissues. BLNPs are composed of a biocompatible lipid bilayer that surrounds the cargo that is designed to be biodegradable, meaning that they can break down into harmless components once they have released their payload (Akbari et al., 2022). There are several advantages to using BLNPs for drug delivery. First, the lipid bilayer can protect the cargo from degradation or clearance by the immune system, allowing for more efficient delivery to the target site. Second, because BLNPs can be designed to be biodegradable, they can be cleared from the body without causing toxicity or other adverse effects. BLNPs can be prepared using various methods, including solvent evaporation, solvent injection, and microemulsion techniques. BLNPs have been studied as a potential delivery system for curcumin to enhance its bioavailability and therapeutic efficacy. Several studies have investigated the use of BLNPs for curcumin delivery. For example, one study reported the preparation of curcumin-loaded BLNPs using a solvent evaporation method. The resulting nanoparticles were found to have a high encapsulation efficiency and a sustained release of curcumin over time, suggesting they could be a promising delivery system for curcumin (Li et al., 2021b).

### 5.3 Carbon complex nanoparticles

Carbon complex nanoparticles (CCNPs) are a type of carbon-based nanoparticle that has been developed for various applications, including drug delivery, imaging, and sensing. CCNPs are typically composed of a core of carbon material, such as carbon nanotubes or graphene, which is coated with a layer of organic or inorganic molecules to improve their properties and functionality (Foldvari and Bagonluri, 2008). One potential application of CCNPs is in drug delivery. CCNPs can be designed to encapsulate various drugs, such as anticancer agents, and can be targeted to specific cells or tissues using surface modifications. The carbon core of the nanoparticle also provides a unique platform for controlled drug release, as the drug can be released in response to changes in temperature, pH, or other environmental factors.

### 5.4 Chitosan-alginate nanoparticles

Chitosan-alginate nanoparticles are a type of biodegradable nanoparticle that can be used for drug delivery, tissue engineering, and other biomedical applications. Chitosan and alginate are both biocompatible and biodegradable polymers that have been widely studied for their potential use in drug delivery and tissue engineering. Chitosan is a natural polymer derived from chitin, a component of crustacean shells, and has been shown to have antimicrobial and immunomodulatory properties. Alginate is a biocompatible polysaccharide derived from brown seaweed that can form hydrogels in the presence of calcium ions. The combination of chitosan and alginate can form nanoparticles with improved stability and drug encapsulation efficiency. One method for producing chitosan-alginate nanoparticles involves ionic gelation, in which the addition of calcium ions cross-links the polymers. Chitosan-alginate nanoparticles can be designed to be mucoadhesive, allowing them to bind to mucosal surfaces and improve drug delivery to the gastrointestinal tract. They can also be modified to target specific cells or tissues, such as tumor cells, by attaching ligands or antibodies to the nanoparticle surface (Li et al., 2022).

### 5.5 Niosomes

Niosomes are a type of lipid nanoparticle that can be used for drug delivery. They are composed of non-ionic surfactants, such as Span and Tween, that form a bilayer structure similar to cell membranes. Niosomes can encapsulate various drugs, including hydrophilic and hydrophobic compounds, and protect the drugs from degradation and clearance by the immune system. Niosomes have several advantages over other drug delivery systems. They are biocompatible, biodegradable, and non-toxic, making them suitable for pharmaceutical and cosmetic applications. They can also be modified to target specific cells or tissues, such as cancer cells, by attaching ligands or antibodies to the noisome surface. Another advantage of niosomes is their ability to improve the bioavailability and pharmacokinetics of drugs (Moghassemi and Hadjizadeh, 2014). The lipid bilayer structure of niosomes allows for the sustained release of drugs over time and can also protect the drugs from degradation in the stomach’s acidic environment.

### 5.6 Silk fibroin nanoparticles

Silk fibroin nanoparticles are biodegradable and biocompatible nanoparticles that can be used for drug delivery, tissue engineering, and other biomedical applications. Silk fibroin is a protein derived from the silkworm cocoon and has been widely studied for its potential use in biomedical applications. Silk fibroin nanoparticles can be produced using various methods, including nanoprecipitation, emulsion/solvent evaporation, and electrospinning. The size, morphology, and surface properties of the nanoparticles can be controlled by adjusting the production parameters. Silk fibroin nanoparticles have several advantages for drug delivery (Mottaghitalab et al., 2015). They are biocompatible and biodegradable and can be designed to release drugs over a sustained period of time. They can also be modified to target specific cells or tissues, such as cancer cells, by attaching ligands or antibodies to the nanoparticle surface.

### 5.7 Polymer-based nanoparticles

Polymer-based nanoparticles are a type of nanoparticle that is composed of synthetic or natural polymers, such as poly (lactic-co-glycolic acid) (PLGA), polyethylene glycol (PEG), chitosan, and polyvinyl alcohol (PVA). These nanoparticles are typically between 10 and 1,000 nm in size and can be used for drug delivery, imaging, and tissue engineering applications. Polymer-based nanoparticles can be synthesized using various methods, including emulsion/solvent evaporation, nanoprecipitation, and electrospinning. The choice of method will depend on the specific polymer used and the desired properties of the nanoparticles. One advantage of polymer-based nanoparticles is their biocompatibility and biodegradability. Polymers such as PLGA and chitosan are approved by regulatory agencies for use in medical devices and drug delivery systems. Polymer-based nanoparticles can be designed to release drugs over a sustained period of time, improving their therapeutic efficacy and reducing side effects (Ayub and Wettig, 2022).

### 5.8 Triblock copolymer-based nanoparticles

Triblock copolymer-based nanoparticles are a type of nanoparticle composed of three blocks of polymers arranged in a linear chain, typically consisting of a hydrophilic block flanked by two hydrophobic blocks. These nanoparticles can be used for drug delivery, imaging, and other biomedical applications. Triblock copolymer-based nanoparticles can be synthesized using various methods, including emulsion/solvent evaporation, nanoprecipitation, and self-assembly. The choice of method will depend on the specific polymer used and the desired properties of the nanoparticles. One advantage of triblock copolymer-based nanoparticles is their ability to self-assemble into well-defined nanostructures. The hydrophobic blocks of the polymer form a core, while the hydrophilic block forms a corona, resulting in a stable and biocompatible nanoparticle. Triblock copolymer-based nanoparticles can be designed to release drugs over a sustained period of time, improving their therapeutic efficacy and reducing side effects. They can also be used to improve the solubility and bioavailability of poorly soluble drugs (Singh et al., 2022).

### 5.9 Dendrosomal nanoparticles

Dendrosomal nanoparticles are a type of nano-carrier composed of dendrimers, which are highly branched, monodisperse macromolecules with a central core and successive layers of branching that can be functionalized with various chemical groups. Dendrimers can be synthesized with a wide range of sizes and surface chemistries, which makes them ideal for designing nanoparticles with precise sizes, shapes, and functionalization for biomedical applications. Dendrosomal nanoparticles are typically prepared by coating the dendrimer with a lipid bilayer or other amphiphilic materials. The resulting nanoparticles are typically between 50 and 200 nm in diameter and can be loaded with drugs, imaging agents, or other payloads for targeted delivery to specific cells or tissues. One advantage of dendrosomal nanoparticles is their biocompatibility and low toxicity, as dendrimers are often composed of natural or biodegradable polymers. Dendrosomal nanoparticles have been shown to have excellent drug loading and release properties and can be modified with targeting ligands to improve their specificity for specific cells or tissues (dos Santos et al., 2022).

Researchers have been intrigued by several categories of nano-carriers, including micelles, nanoparticles, nanoemulsions, nanocrystals, and nanoliposomes, in order to solve issues associated with curcumin’s low bioavailability and pharmacokinetics. In recent years, research has been conducted to determine the efficacy of nanoformulations for improved therapeutic action. Joseph et al., experimented with the diffusion and distribution of curcumin-loaded PLGA-PEG nanoparticles in the brain of newborn rats. Injured areas of the neonate rats demonstrated neuroprotection against a compromised blood–brain barrier. The brain parenchyma allows these nanoparticles to spread effectively (Joseph et al., 2018). Zhang et al. (2018) developed curcumin-loaded polysorbate 80 modified cerasome nanoparticles and used ultrasound-targeted microbubble disruption of the BBB to deliver them to MPTP-induced Parkinson’s disease in rats. This study found that nanoformulations of curcumin had superior stability, longer duration of action, and higher absorption than free curcumin.

**FIGURE 4:**
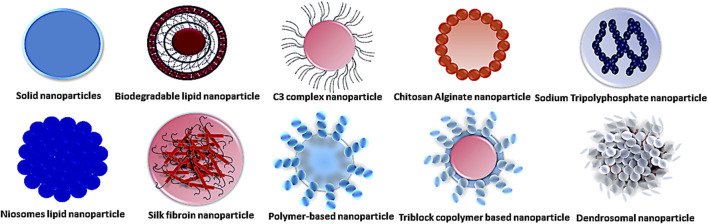
Representing different types of nanoparticle carrier systems carries active pharmaceutical ingredients that have been used in several diseases.

Furthermore, after the administration of created NPs (15 mg curcumin/kg), they reported improved behavior as well as dopamine reduction in experimental animals (Zhang et al., 2018). Polymeric NPs encapsulating curcumin were found to be more effective in shielding neurons from oxidative insults in both *in-vitro* and *in-vivo* investigations. Human SK-N-SH cells were protected from H_2_O_2_-mediated free radicals assaults by nano curcumin therapy. In athymic mice, intraperitoneal administration of nano curcumin (25 mg/kg, twice daily) resulted in considerable curcumin levels in the brain, as well as lower levels of H_2_O_2_ and caspase activity and higher glutathione concentrations (Ray et al., 2011). Curcumin-encapsulated PLGA NPs were found to have an improved effect on the proliferation and differentiation of neural stem cells *in-vitro* and *in-vivo* than pure curcumin in a study. In the hippocampus area, the encapsulated NPs greatly increased the gene expression associated with proliferation and neural development. In the rat model of Aβ-induced AD, NPs administration showed an improved effect on reversing the altered learning and memory characteristics. These NPs stimulated neurogenesis via the canonical Wnt/β-catenin pathway, according to *in silico* molecular docking studies (Tiwari et al., 2014). Mathew et al. (2012) developed PLGA-coated curcumin NPs that were water soluble and combined with Tet-1 peptide, a peptide with increased affinity for neurons. They discovered that curcumin-encapsulated NPs effectively eliminated amyloid aggregates and had antioxidant and non-cytotoxic properties (Mathew et al., 2012). Some of the nanoparticulate systems used for encapsulating curcumin, are shown in Figure 4, and the list of the nanoformulations of curcumin in several neurological disorders, is presented in Table 4.

**TABLE 4:** Studies showing the neuroprotective activity of curcumin nanoformulations in different experimental models.

S.No.	Nanoformulation	Size	Experimental model	Proposed mechanism	Reference
1	Biodegradable lipid-based discoidal NPs	10–25 nm	Spinal cord injury in rats	Encapsulated curcumin caused downregulation of the IL-11 and IL-1β, insignificant downregulation of TNF-α, IFN-γ, IL-6, and RANTES, suppression of reactive astrocytes activation, upregulation of IL-10 and IL-13 (anti-inflammatory cytokines), a higher quantity of newly sprouted axons	Krupa et al. (2019)
2	Solid lipid NPs	NA	Epileptic mice	Curcumin-encapsulated NPs improved behavioral performance, more NeuN in the hippocampus, and reduced neuronal apoptosis via Bcl2 and P38 MAPK signaling	Huang et al. (2020)
3	C3 complex NPs	NA	PTZ-induced seizures in mice	Nanocurcumin treatment increased the seizure threshold and downregulated NO.	Aminirad et al. (2017)
4	Chitosan-Alginate- Sodium tripolyphosphate NPs	NA	PTZ-induced kindling in mice	Curcumin-loaded NPs alleviated neuronal cell death, upregulated the EPO and Klotho levels, reduction of mRNA expression of TNF-α in the hippocampus	Mansoor et al. (2018)
5	Chitosan-Alginate- Sodium tripolyphosphate NPs	NA	PTZ-induced kindling in mice	Curcumin NPs showed anticonvulsant activity, prevented cognition impairment, and reduced glial activation and cell death	Hashemian et al. (2017)
6	Niosomes NPs	60 nm	Traumatic brain injury in rats	hNS/PCs + Curcumin-loaded NPs showed improved locomotor activity, improved brain edema, decreased astrogliosis, and decreased TLR4, NF-κB-, and TNF-α- positive cells	Narouiepour et al. (2022)
7	NH_2_-PEO-PCL NPs	99.5 + 7.3 nm	Parkinson’s disease (Vero and PC12 cells)	Cells treated with varied concentrations of L-DOPA and curcumin-loaded NP for up to 72 h retained a high percentage of vitality	Mogharbel et al. (2022)
8	Silk fibroin NPs	150 nm	Friedrich’s ataxia in mice	Removed iron from the heart, reduced oxidative stress, potentiated iron-sulfur cluster biogenesis, improved morphology and function of mitochondria, improved behavior scores	Xu et al. (2022)
9	Polymer (PEG)-based NPs	127.31 ± 2.73 nm	Intracerebral hemorrhage model in C57BL/6 mice	Attenuation of neuronal damage, suppression of iron deposition in the brain tissue around the site of hematoma, elevated perihematomal GPX4 expression by regulating HMOX1 (HO-1) and NFE2L2 (NRF2) expression	Yang et al. (2021)
10	Triblock copolymer nano micelles	96.67 ± 11.94 nm	BCCAO stroke model in rats	Higher bioavailability in plasma, heart, brain, and kidneys, downregulated expressions of IL-1β, NF-κB, IL-6, and TNF-α, and a significant reduction in lipid peroxidation	Li et al. (2021c)
11	Dendrosomal NPs	NA	Cuprizone-induced demyelination in mice	Inhibited accumulation of microglia and astrocytes and in the corpus callosum, a higher index of myelin basic protein intensity in the corpus callosum (indicative of myelin content)	Motavaf et al. (2020)
12	Pluronic F127 NPs	∼11 nm	*In vitro*: BBB model *In vivo*: Healthy mice	Encapsulated NPs showed more vigorous fluorescent intensity (6.5 folds) in the brain as compared with pure curcumin. Encapsulated curcumin kept its capacity to bind with Aβ plaques and presented better antiapoptotic activity and improved redox balancing mechanism as compared to pure curcumin	Singh et al. (2020)

**Abbreviations: PTZ,** Pentylenetetrazole; **NPs,** Nanoparticles; **IL-11,** Interleukin-11; **IL-1β,** Interleukin-1β; **TNF-α,** Tumour necrosis factor-α; **IL-6,** Interleukin-6; **IFN-γ,** Interferon-γ; **RANTES,** Regulated on activation, normal T cell expressed and secreted; **IL-10,** Interleukin-10; **IL-13,** Interleukin-13; **Bcl-2,** B-cell lymphoma 2; **MAPK,** Mitogen-activated protein kinase; **NO,** Nitric oxide; EPO, erythropoietin; **hNS/PCs,** human neural stem/progenitor cells; **TLR4,** Toll-like receptor 4; **NF-κB,** Nuclear factor-κB; PEO, Poly (ethylene oxide); **PCL,** Poly (ε-caprolactone); **PEG,** Polyethylene glycol; **BCCAO,** Bilateral common carotid artery occlusion.

## 6 Conclusion

The current review mainly dissected modulatory effects of curcumin on epileptic pathophysiology. Curcumin impose multiple immunomodulatory effects by modulating immune molecules, including T- and B-lymphocytes, macrophages, cytokines, chemokines, DCs, and transcription factors, thus elicits anti-inflammatory mechanism. Curcumin acts by inhibiting NF-κB transcription, which is responsible for the production of cytokines such as IL-6, IL-12, and TNF-α. It is also suppressing NF-κB signaling by imposing an inhibitory action on BLYS, which is an important innate inflammatory response in epilepsy patients. Additionally, curcumin downregulates mTOR signaling which eventually causes a reduction in the levels of cytokines such as COX-2, and IL-6 which are upregulated in epileptic subjects. Curcumin administration suppresses glial activation, a common finding in epileptic patients, by suppressing the levels of cytokines (IL-6, IL-1β, and, TNF-α) and chemokines (MCP-1). Also, curcumin administration reduces the expression of MHC-II and co-stimulatory molecules like CD80, CD40, and CD205. Curcumin activates Nrf2, a regulator for mitigating oxidative stress, and reduces oxidative damage by inhibiting the activation of NF-κB. Curcumin reduces the markers of lipid peroxidation and increases the levels of antioxidant molecules like GSH in epileptic brains. Curcumin prevents the release of cytochrome C from mitochondria and inhibits the formation of apoptosomes and the activity of caspases. The antiapoptotic, antioxidant, and immune modifying abilities of curcumin are the reported mechanisms responsible for enhancing cognition, memory processes, and neurobehavior. Curcumin is a potent HDAC inhibitor, and HDAC inhibition is an emerging therapeutic approach for epilepsy. Also, curcumin increases the activation of histone H4.

Epigenetic associated hypermethylation in TLE patients reversed upon curcumin administration. To overcome the poor bioavailability and physic-chemical properties of curcumin, nanonization has emerged as a promising strategy in several neurological disorders. Several studies have reported better pharmacological efficacy and molecular interactions than pure curcumin. The current literature supports curcumin administration as an adjuvant therapy to the conventional AEDs for treating epilepsy. More combinatorial studies are warranted to understand the synergistic effect imposed by the curcumin. Still many preclinical are in the waiting list to reach the human clinical trials. Therefore, the findings from the reported preclinical research results are essential and may be the scaffold for evaluation of curcumin-based nanoformulation and its pure form for their anti-epileptic potential in clinical studies.
